# Climate change vulnerability assessment of the main marine commercial fish and invertebrates of Portugal

**DOI:** 10.1038/s41598-021-82595-5

**Published:** 2021-02-03

**Authors:** Juan Bueno-Pardo, Daniela Nobre, João N. Monteiro, Pedro M. Sousa, Eudriano F. S. Costa, Vânia Baptista, Andreia Ovelheiro, Vasco M. N. C. S. Vieira, Luís Chícharo, Miguel Gaspar, Karim Erzini, Susan Kay, Henrique Queiroga, Maria A. Teodósio, Francisco Leitão

**Affiliations:** 1 Centro de Ciências do Mar (CCMAR), Universidade do Algarve, Campus de Gambelas, 8005-139 Faro, Portugal; 2 Maretec, Instituto Superior Técnico, Universidade de Lisboa, Av. Rovisco Pais, 1049-001 Lisboa, Portugal; 3 CIMA, Faculdade de Ciência e Tecnologia, Universidade do Algarve, Campus de Gambelas, 8005-139 Faro, Portugal; 4 Centro de Olhão, Instituto Português do Mar e a Atmósfera (IPMA), 8700-305 Olhão, Portugal; 5 Plymouth Marine Laboratory, Prospect Place, The Hoe, Plymouth, PL1 3DH UK; 6 Departamento de Biologia e Centro de Estudos do Ambiente e do Mar (CESAM), Universidade de Aveiro, Campus Universitário de Santiago, 3810-193 Aveiro, Portugal

**Keywords:** Environmental impact, Climate-change ecology, Ecosystem services, Marine biology

## Abstract

This is the first attempt to apply an expert-based ecological vulnerability assessment of the effects of climate change on the main marine resources of Portugal. The vulnerability, exposure, sensitivity, adaptive capacity, and expected directional effects of 74 species of fish and invertebrates of commercial interest is estimated based on criteria related to their life-history and level of conservation or exploitation. This analysis is performed separately for three regions of Portugal and two scenarios of climate change (RCP 4.5 and RCP 8.5). To do that, the fourth assessment report IPCC framework for vulnerability assessments was coupled to the outputs of a physical-biogeochemical model allowing to weight the exposure of the species by the expected variability of the environmental variables in the future. The highest vulnerabilities were found for some migratory and elasmobranch species, although overall vulnerability scores were low probably due to the high adaptive capacity of species from temperate ecosystems. Among regions, the highest average vulnerability was estimated for the species in the Central region while higher vulnerabilities were identified under climate change scenario RCP 8.5 in the three regions, due to higher expected climatic variability. This work establishes the basis for the assessment of the vulnerability of the human activities relying on marine resources in the context of climate change.

## Introduction

Portugal has the third highest level of fish consumption per capita in the world. The Portuguese fisheries industry represents 0.24% of the gross domestic product of the country^1^, although it can reach much higher relevance at a local scale^2^. The number of registered fishermen in 2018 was 16,164 and the total revenue of marine landings was 291,715 10^3^ €^3^. As in other primary sectors, provisioning from marine fisheries varies because of both environmental and social fluctuations^4^. The current fast rate of environmental change induced by human activities challenges, as never before, the capacity of response of the sector by disrupting the ecological equilibrium of the underlying marine ecosystem^5^.

**Figure 1:**
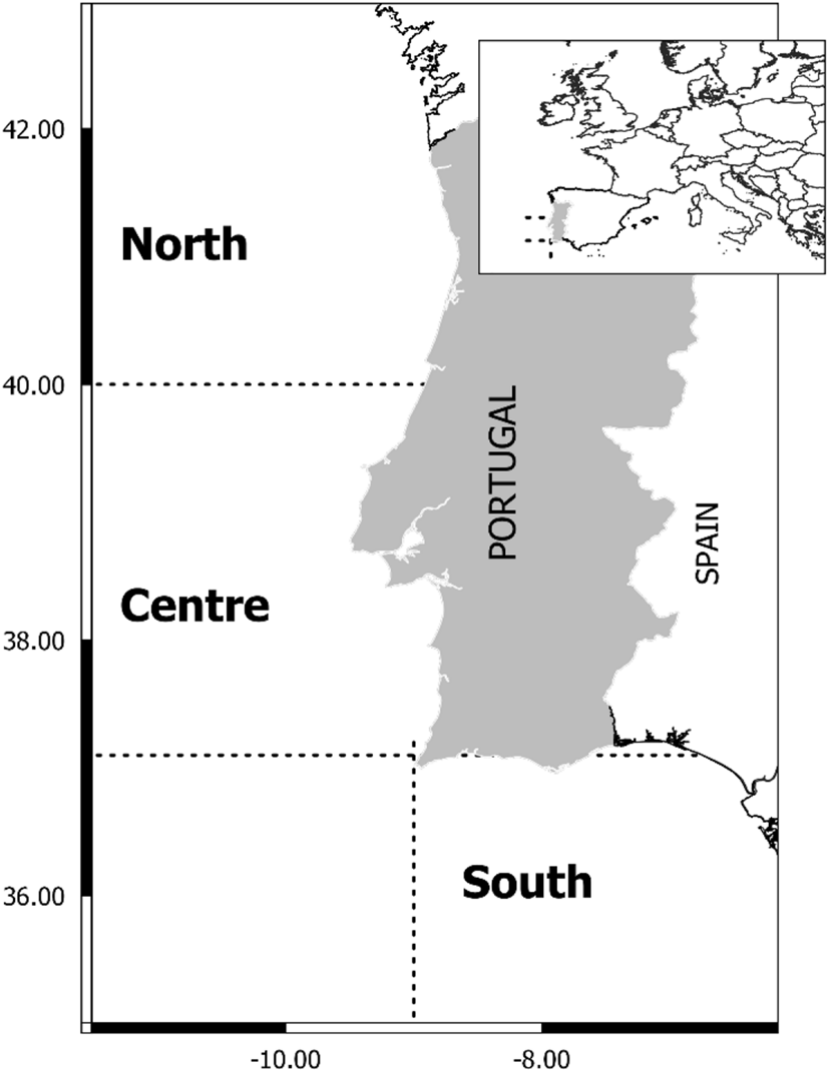
Map of Portugal showing the regions of study. The map was created using QGIS 3.10 (http://qgis.org).

The coast of Portugal follows a North–South orientation and is situated in a continental west boundary at temperate latitudes (37–42° N) (Fig. 1). As a result, the marine ecosystem of Portugal is strongly influenced by upwelling events^6^, while showing strong latitudinal environmental gradients^7^. The likely consequences of climate change on upwelling systems have been broadly discussed (e.g. Refs.^8,9^). Although the high number of variables and their interplay makes it difficult to predict the response of these ecosystems, it has been broadly accepted that an increase of spring and summer upwelling events are expected in the next years as a consequence of increased thermal difference between the land and ocean surfaces, promoting the intensification of upwelling-favourable winds and offshore advection^8^. In Portugal, between 1950 and 2010, the coastal temperature increased at a rate of + 0.1 °C decade^−1^ in the Northwestern and Southwestern coasts and + 0.2 °C decade^−1^ in the Southern coast^10^ which points to the necessary to consider the inherent impacts of climate change along coastal areas in studies on ecosystem productivity or fisheries (e.g. Ref.^4^). The frequency and intensity of upwelling events also increased in recent years in Portugal^6^ (but see Ref.^11^ for an opposite trend^12^). These environmental changes are likely to lead to consequences for the main fisheries of Portugal, including changes in the catch composition by the introduction of subtropical species^13^, or fluctuations of landings due to environmental changes with mechanistic consequences on the recruitment of small and medium pelagics^14–18^ also found a general decreasing trend of landings of species with affinity for temperate waters and an opposite trend for species with affinity for subtropical/tropical waters, evidencing that species might respond differently to climate change due to their ecology and biology. A big effort to understand the effect of environmental variables on marine fish landings has been paid in recent years (e.g. Refs.^4,19,20^). These works relate observed fish landings to environmental time series and usually lack a mechanistic understanding of the relationship between environment and (i) organisms ecology or (ii) fisheries functioning, being limited to inferring the evolution of these fisheries under future scenarios of climate change.

To evaluate the potential risks posed by climate change on marine species, two strategies are commonly used. The first one combines population models with climate models to develop mechanistic frameworks that are projected into the future using expected future environmental parameters as driving forces (e.g. range of models in Ref.^21^). These approaches are validated using environmental conditions from the past, allowing to hindcast the population dynamics and compare them with population observations^22^. The second perspective considers trait-based climate change vulnerability assessments to identify the most vulnerable parts of marine systems, including both exploited organisms and exploiting communities (e.g. Refs.^23,24^). This method often relies on expert judgement to estimate characteristics of the species related to their vulnerability, such as the level of exposure to environmental change, or their resilience^25^. In this context, Cheung et al.^26^ developed a fuzzy logic expert system for estimating intrinsic extinction vulnerabilities of seamount fishes related to fishing. This system evaluates certain life-history traits that are allocated into domains defined by membership functions. The benefit of this methodology is that it can reach conclusions from premises with a gradation of truth, instead of classifying them as true or false. Life history traits can thus be assigned to bins representing ranges of values into which species can be allocated, allowing to add some uncertainty in the classification^23,27,28^. Similarly, the Intergovernmental Panel for Climate Change (IPCC) of the United Nations developed a conceptual framework for the assessment of the vulnerability of ecosystems, communities, and species to the impact of climate change (climate change vulnerability assessments)^29^. This methodology is based on the definition of different criteria relating to the exposure, sensitivity, and adaptive capacity of the subject of study to environmental change. Due to the simplicity of the framework, it has been widely used by conservation managers to estimate the climate change vulnerability of specific habitats (e.g. Ref.^30^), groups of species (e.g. Ref.^31^), and human communities (e.g. Ref.^32^). Including human communities into these vulnerability assessments has been recently found to be a valuable tool for defining actions of anticipation and mitigation in the context of climate change^33–35^. This approach integrates the vulnerability of human communities to the changes imposed on the ecosystem by environmental change. Cinner et al.^36^, extended the conceptual framework developed by the IPCC to assess the vulnerability of coral reef fisheries considering that the ecological vulnerability of the targeted species represents the exposure of the human community to climate change (Fig. 1 in Cinner et al.^36^) and assessing their sensitivity and adaptive capacity on the basis of social and economic criteria.

Here we aim at setting the basis for the assessment of the vulnerability of Portuguese fisheries by assessing the vulnerability to climate change of the main fish and invertebrates of ecological and economical interest in the country. Our perspective is broad and comprises many different aspects of the ecology and biology of the organisms under consideration, in regards of their different biological aspects and stages of their life cycles (involving very different environments in most cases). This wide approach poses a challenge as the information on the biology and ecology of the initial stages of life is scarce and difficult to obtain in comparison to adult data. The mechanisms underlying the survival of eggs and larvae in the marine system are complex, with many interrelated variables playing very different roles^37^. Also, the consequences of climate change in the upwelling-driven marine ecosystem mentioned above are still poorly understood at the biological scale^38,39^. For example, the increase of winds promoting upwelling could be beneficial for organisms with planktonic larvae as the upwelling of deep nutrient-rich water to the surface could promote primary production and hence favour larvae survival. But on the other hand, the increase of upwelling could be prejudicial as stronger upwelling events could wash away larvae from the coast, causing massive deaths and pervading recruitment back to coastal areas^40^. Another example is the different effect of the temperature increase on organisms with planktonic larvae. Higher temperature shortens the developmental time of organisms implying that planktonic larvae have less time to disperse. This would have a negative effect on the connectivity between populations but, at the same time, increased temperature could contribute positively to the fecundity of females^41^. A higher number of dispersive particles would compensate for the shorter time these particles have to reach new environments, balancing the final effect on the connectivity between populations. The application of fuzzy theory^26^ on ecological risk assessment allows to account for all this uncertainty as different life-history characteristics can be classified by multiple categories with different degrees of membership, and the different aspects behind a given ecological trait can be treated separately.

The separated treatment of the dimensions of vulnerability (exposure, sensitivity, and adaptive capacity, according to IPCC’s fourth assessment report^29^) allows to introduce the effect of different degrees of environmental variation in the assessment. In this regard, considering different levels of exposure related to different regions or different scenarios of climate change allows us to obtain specific degrees of vulnerability. Here we consider three regions of Portugal (North, Centre, South; Fig. 1) and two IPCC scenarios: RCP 4.5 and RCP 8.5. For each combination of region and scenario, the level of exposure (E), sensitivity (S) and adaptive capacity (AC) is assessed by a panel of experts, considering different life-history or fisheries-related indicators ranked in three categories following an expert-based distribution of certainty. The establishment of a ranking of vulnerabilities of the main Portuguese marine resources represents valuable information to establish priorities of protection and as a first step to assess the ecological exposure of the human communities depending—directly or indirectly—on these organisms for subsisting.

## Materials and methods

### Selection of species

The list of species for the vulnerability assessment was based on five different criteria. First, we considered the proportion of each species in the total Portuguese landings between 1989 and 2015, using public landings data from the Direção Geral dos Recursos Marinhos de Portugal (DGRM). The most landed species, accounting for 95% of purse seine, 70% of trawling and 70% of the multigear landings, were included. This selection was carried out separately for each combination of gear and region (Supplementary Table SI1-1). Second, species were chosen in regards of their economic relevance, considering the species representing more than 3% of the total economic revenue of the marine landings within each combination of region and gear (DGRM, Supplementary Table SI1-2). Third, we included the most frequent species in the discards of Portuguese fisheries, according to the work of Leitão et al.^42^, where the top-ten discarded species per *métier* are listed (Supplementary Table SI1-3). Fourth, we included the species of importance for the canning industry, obtained by means of a survey covering the main can enterprises of Portugal (Supplementary Table SI1-5). Fifth, a selection of the species of relevance for the Moroccan fisheries sector was carried out, using the reports from the Department of Marine Fisheries of the Kingdom of Morocco^43^ and the FAO software FishStatJ (most captured species between 2007 and 2017^44^) (Supplementary Table SI1-6). Additionally, due to their importance for specific fleet segments, we included some shark species of interest that were not included by the previous criteria. The selection of shark species was based on reports from the Instituto Português do Mar e as Pescas (IPMA) and included: *Galeus melastomus, Prionace glauca, Squalus acanthias, Scyliorhinus canicula,* and *Hexanchus griseus*. Some riverine species were finally removed from the list (*Petromyzon marinus*, *Salmo trutta*), as well as cod (*Gadus morhua*), since it is not captured within the area of study. Finally, some extra species were pointed out by experts during the evaluation process as species with economic interest (*Pollicipes pollicipes*) or with potential distribution shift into/from the area of study in the context of climate change such as the bivalves *Callista chione* and *Ruditapes philippinarum*, and the crabs *Callinectes sapidus* and *Carcinus maenas*. The final list of species considered, and their functional group are shown in Table 1.

**Table 1:** Species and functional groups considered during the climate change vulnerability assessment.

Functional group	Species number	Species	Functional group	Species number	Species
Cephalopods	1	*Sepia officinalis*	Large flatfishes	38	*Scophthalmus maximus*
	2	*Octopus vulgaris*	Medium flatfishes	39	*Scophthalmus rhombus*
	3	*Loligo vulgaris*		40	*Solea solea*
	4	*Illex coindetii*	Large pelagics	41	*Sarda sarda*
Large bathydemersals	5	*Lepidopus caudatus*		42	*Thunnus thynnus*
	6	*Lophius piscatorius*	Medium pelagics	43	*Trachurus trachurus*
Medium bathypelagics	7	*Brama brama*		44	*Scomber scombrus*
Large benthopelagics	8	*Aphanopus carbo*		45	*Scomber colias*
	9	*Salmo salar*		46	*Belone belone*
Medium benthopelagics	10	*Micromesistius poutassou*	Small pelagics	47	*Sardina pilchardus*
	11	*Trisopterus luscus*		48	*Engraulis encrasicolus*
	12	*Trachurus picturatus*		49	*Sardinella spp.*
	13	*Pagellus erythrinus*	Large rays	50	*Raja clavata*
	14	*Spondyliosoma cantharus*	Large sharks	51	*Centroscymnus coelolepis*
	15	*Diplodus vulgaris*		52	*Prionace glauca*
	16	*Pagellus acarne*		53	*Squalus acanthias*
	17	*Lithognathus mormyrus*		54	*Scyliorhinus canicula*
	18	*Chelon auratus*		55	*Hexanchus griseus*
Large demersals	19	*Merluccius merluccius*	Crabs	56	*Callinectes sapidus*
	20	*Conger conger*		57	*Necora puber*
	21	*Dicentrarchus labrax*		58	*Maja squinado*
	22	*Anguilla anguilla*		59	*Carcinus maenas*
Medium demersals	23	*Sparus aurata*	Lobsters	60	*Homarus gammarus*
	24	*Mullus surmuletus*		61	*Palinurus elephas*
	25	*Diplodus sargus*		62	*Nephrops norvegicus*
	26	*Chelidonichthys lucerna*	Shrimps	63	*Parapenaeus longirostris*
	27	*Umbrina cirrosa*		64	*Aristeus antennatus*
	28	*Boops boops*		65	*Palaemon serratus*
	29	*Trachinus draco*	Bivalves	66	*Callista chione*
	30	*Chelidonichthys obscurus*		67	*Cerastoderma edule*
	31	*Scorpaena notata*		68	*Ruditapes decussatus*
	32	*Halobatrachus didactylus*		69	*Ruditapes philippinarum*
	33	*Cynoscion regalis*		70	*Spisula solida*
Small demersals	34	*Microchirus azevia*		71	*Ensis siliqua*
	35	*Macroramphosus scolopax*		72	*Donax trunculus*
	36	*Capros aper*		73	*Mytilus galloprovincialis*
	37	*Spicara maena*	Barnacles	74	*Pollicipes pollicipes*

### Environmental change

RCP (representative concentration pathway) scenarios of atmospheric greenhouse gas concentration have been proposed by the IPCC for use in research to project the evolution of environmental variables. Using scenarios RCP 4.5 and RCP 8.5 (predicting a global warming of 1.8 and 3.7 °C respectively by the end of the twenty-first century) as forcing, the POLCOMS-ERSEM model^45^ forecasted a wide array of physical, chemical and biological variables for the Northeast Atlantic and adjacent seas at a resolution of 0.1 degree (approximately 11 km). For the evaluation of the vulnerability of the species of interest, a selection of the most cited variables with impact on the ecology of marine organisms in the Portuguese marine environment was carried out (e.g. Refs.^7–9^). As a result, these variables were finally considered: sea surface temperature (SST, °C), surface pH, surface salinity (psu), surface zooplankton biomass (mol m^−3^), surface phytoplankton biomass (mol m^−3^), surface northward and eastward current velocities (m s^−1^) and river discharge (m^−3^ year^−1^). The zooplankton and phytoplankton biomass were summed to obtain an overall plankton biomass (mol m^−3^) which was finally used in the assessment of vulnerability. Surface variables were calculated using the top sigma layer of the outputs of the model.

Two time slices of the POLCOMS-ERSEM outputs were used to define two periods for comparison. The first was between 2000 and 2019 setting a reference point for the state of the environment at the beginning of the century (hereafter “reference”), then, the period between 2040 and 2059 served to define the likely state of the environment in the near future (hereafter “future”). Defining the future and reference periods allowed us to compare the expected degree of change of the environmental variables between both periods. To do this on a regional basis, we considered the outputs of the model for each region of Portugal (North, Centre, and South; Fig. 1) and calculated a dimensionless variation index (VI) using the mean of each variable during the reference and future periods, and the standard deviation of the reference period:

1 $$ {\text{VI}} = \frac{{\left( {\mu \,future - \mu \,reference} \right)}}{\sigma \,reference} , $$

where *µ future* and *µ reference* represent the regional average values of the corresponding time slice of the variable, and *σ reference* is the standard deviation of the regional values in the reference time slice (except for the variable river discharge, for which the average and standard deviation are calculated on a temporal basis) VI takes theoretical values between 0 (when there is no variation between future and reference) and ± infinite (when reference shows no variation all over the region of study). VI was used to weight the influence of each variable in the assessment of the exposure of the species to climate change n Table 2. The idea was to capture the degree of variability of each physical variable, so species exposed to the most variable environmental conditions would be more exposed to the effects of climate change. Then, a weight factor was calculated normalizing between 1 and 2 the absolute values of the VI defined above (“weight factor 1” in Table 2).

**Table 2:** Expected physical variability between 2000–2019 (reference) and 2040–2059 (future) according to POLCOMS-ERSEM physical-biogeochemical model. Outputs are shown considering three regions of Portugal (North, Centre, South) and two scenarios of climate change (RCP 4.5 and RCP 8.5). Weight factor 1 captures the degree of variability of the physical variables (see “Methods”). Weight factor 2 represents the likely impact on the physiology of the marine organisms and was obtained from the experts’ criteria. The final weight factor, used in the vulnerability assessment, is the average between weight factor 1 and 2.

Variable	Region	Scenario	Average reference	Average future	SD reference	VI	Weight factor 1	Weight factor 2	Final weight factor
SST (°C)	North	RCP 4.5	16.2109	16.1187	0.4237	− 0.21	1.0248	1.8	1.4124
		RCP 8.5		16.1634		− 0.11	1.0118	1.8	1.4059
	Centre	RCP 4.5	17.3394	17.5855	0.5673	+ 0.43	1.0514	1.8	1.4257
		RCP 8.5		17.6430		+ 0.53	1.0639	1.8	1.4319
	South	RCP 4.5	19.1247	19.4834	0.4566	+ 0.78	1.0947	1.8	1.4473
		RCP 8.5		19.8362		+ 1.55	1.1898	1.8	1.4948
Salinity (PSU)	North	RCP 4.5	35.3938	35.0774	0.1574	− 2.01	1.2453	1.2	1.2226
		RCP 8.5		34.9138		− 3.04	1.3731	1.2	1.2865
	Centre	RCP 4.5	35.7099	35.5385	0.0403	− 4.24	1.5207	1.2	1.3603
		RCP 8.5		35.3814		− 8.14	2	1.2	1.6000
	South	RCP 4.5	35.8391	35.7443	0.1094	− 0.86	1.1046	1.2	1.1523
		RCP 8.5		35.6166		− 2.03	1.2482	1.2	1.2240
pH	North	RCP 4.5	8.1784	8.1407	0.0254	− 1.47	1.1801	1	1.0900
		RCP 8.5		8.1406		− 1.48	1.1804	1	1.0902
	Centre	RCP 4.5	8.2380	8.1823	0.0092	− 6.02	1.7399	1	1.3699
		RCP 8.5		8.1715		− 7.19	1.8835	1	1.4417
	South	RCP 4.5	8.2290	8.1903	0.0108	− 3.55	1.4358	1	1.2179
		RCP 8.5		8.1807		− 4.44	1.5446	1	1.2723
Eastward current (m s^−1^)	North	RCP 4.5	-0.0066	− 0.0098	0.0211	− 0.15	1.0166	2	1.5083
		RCP 8.5		− 0.0185		− 0.56	1.0671	2	1.5335
	Centre	RCP 4.5	-0.0152	− 0.0206	0.0156	− 0.35	1.0411	2	1.5206
		RCP 8.5		− 0.0165		− 0.08	1.0085	2	1.5043
Northward current (m s^−1^)	South	RCP 4.5	0.0052	0.0071	0.0473	− 0.25	1.0028	2	1.5014
		RCP 8.5		0.0035		− 0.03	1.0027	2	1.5013
Plankton concentration (mol m^−3^)	North	RCP 4.5	0.0099	0.0112	0.0031	+ 0.40	1.0472	1.6	1.3236
		RCP 8.5		0.0116		+ 0.52	1.0625	1.6	1.3312
	Centre	RCP 4.5	0.0086	0.0095	0.0027	+ 0.31	1.0362	1.6	1.3181
		RCP 8.5		0.0102		+ 0.54	1.0647	1.6	1.3324
	South	RCP 4.5	0.0064	0.0067	0.0019	+ 0.164	1.0177	1.6	1.3088
		RCP 8.5		0.0067		+ 0.13	1.0152	1.6	1.3076
River flow (m^−3^ s^−1^)	North	RCP 4.5	734,209	761,171	181,656	+ 0.14	1.0163	1.4	1.2082
		RCP 8.5		731,350		− 0.01	1	1.4	1.2000
	Centre	RCP 4.5	144,533	162,081	72,258	+ 0.24	1.0279	1.4	1.2140
		RCP 8.5		140,402		− 0.05	1.0051	1.4	1.2025
	South	RCP 4.5	255,960	296,071	97,765	+ 0.41	1.0485	1.4	1.2243
		RCP 8.5		243,390		− 0.12	1.0139	1.4	1.2069

Since two versions of the future period were available (climate change scenarios RCP 4.5 and RCP 8.5), the level of exposure to changing environmental variables was calculated separately for both climate change scenarios, making it possible to estimate the overall vulnerability of the species under each scenario separately.

Beyond the degree of variability of each variable, a panel of experts on the ecology of marine organisms of Portugal was asked to rank, according to the likely impact on the physiology of marine organisms, the physical variables under consideration. Each expert was asked to order the variables independently, but a consensus answer was finally asked from them. The ranking of the physical variables was posteriorly transformed numerically between 1 and 2, being 1 the less relevant variable and 2 the most relevant variable. Intermediate variables got a value between 1 and 2 following equally distanced steps (see “weight factor 2” in Table 2). The final weight given to each physical variable during the vulnerability assessment was calculated as the average between weight factors 1 and 2 (“final weight factor” in Table 2). It was possible to estimate this parameter for all the exposure indicators with exception of the extreme events frequency, which was not included in the POLCOMS-ERSEM outputs. The likely evolution of this parameter is controversial and thus, a final weight factor of 1 was assigned by consensus with the panel of experts. In the case of oceanic currents, considered as a proxy for upwelling, we considered eastward currents in the North and Centre regions (North–South oriented coast) and northward currents in the South region (East–West oriented coast).

### Vulnerability assessment

#### Indicators

The vulnerability of the species to climate change was evaluated following the conceptual framework described in the 4th Assessment Report of the IPCC^29^. This approach assumes that the vulnerability (V) of species to environmental change is a function of: (1) their exposure (E) to the changing environmental variables (defined as the overlap between the expected geographic range of change of the variables and the area/habitats of occurrence of a given species), (2) their sensitivity (S) to environmental change (considered as the degree to a which extent a given species will be affected—in terms of population dynamics or life-history traits—by a change in the environment), and (3) their adaptive capacity (AC) to environmental change (understood as the mechanisms of a given species to resist to a specific change of the environment and recover to the state prior to the perturbation).

For each species, the degree of exposure, sensitivity and adaptive capacity was evaluated considering different aspects (hereafter “indicators”) of its biology, ecology, and exploitation (see Supplementary SI2 for a description of the indicators). The selection of the indicators was made considering the context of climate change in the Portuguese marine environment. Hence, for the level of exposure, the most referenced environmental variables with impact on the ecology of the species of interest were chosen. For the analysis of the sensitivity, a selection of life history traits driving the relationship between the species’ population dynamics and the environment was carried out based on existing literature (e.g. Refs.^23,26,28,36^). The traits finally considered were: trophic level, fecundity, number of reproductive events in a lifetime, egg spawning strategy, individual growth parameters (growth coefficient, *k*, in Von Bertalanffy’s growth function), age at maturity, longevity, intrinsic population growth rate (*r*), sexual strategy (gonochorism, hermaphroditism or protogyny/protandry), length of the spawning seasons, planktonic larval duration (PLD), latitudinal range of distribution, temperature range of distribution, adult mobility, seasonal migrations, sociability, and complexity of the reproductive strategy. The adaptive capacity of the species was analysed considering different aspects related to the degree of conservation or exploitation of the species and the kind of fisheries associated, which give an idea of the capacity of response of the populations to environmental change at a national or regional scale. In this case we considered: the ICES stock status (referred to Portuguese or Iberian stocks when available), the general replenishment potential of the species, related to different life-history parameters such as growth and reproduction, the vulnerability degree assigned by the IUCN, the specific vulnerability to fisheries assessed in Cheung et al.^26^, and the fishing pressure suffered by each species in Portuguese waters.

#### Expert’s assessment

To evaluate each species from the point of view of each indicator, a fuzzy logic expert-judgement method was applied^26^. This method consists of categorizing the range of possible answers or values of each indicator into three levels (bins) corresponding to low, moderate, or high levels of exposure, sensitivity and adaptive capacity, respectively. The number of levels considered (3) has been found to be sufficient for this kind of study^28,46^, and their ranges were defined for each indicator following the existing literature, adjusting their values to the reality of the Portuguese marine environment. For a description of the levels within each indicator see Supplementary SI2.

Assigning each species to each bin of each indicator was carried by a group of experts in marine biology and ecology with experience in the Portuguese marine environment. A variable number of species was assigned to each expert in regards of their field of knowledge and previous experience. Each species received a minimum of three experts and a maximum of four. The number of tallies assigned to each bin of each indicator (variable between 0 and 5) represented the degree of confidence in the answer. In this way, an absolute confidence in the answer provided was represented by allocating 5 tallies in the corresponding bin, while spreading the five tallies among the three bins meant the highest level of uncertainty. In order to avoid biases in the expert evaluations, each expert was provided with the description of the indicators and their bins found in Supplementary SI2, the maps of climate variability found in Supplementary SI3, and a list of online resources to consult. The experts were allowed to consult any other scientific literature for their evaluations if needed.

After the evaluation of each indicator of exposure, sensitivity and adaptive capacity, each expert was asked to provide a formed opinion on the likely direction of the effects of climate change for each species. This directional effect (DE) evaluation had two steps: (1) the allocation of five tallies among three bins representing negative, neutral, or positive DE, and (2) providing a short rationale text explaining the allocation of tallies among the bins.

Experts were also asked to score the quality of the data used to distribute the tallies among the bins of each indicator following the methodology of Hare et al.^23^. In this case, the experts should assign a value between 0 and 3 to describe the quality of the information. These values correspond to (0) No Data. No information is available to provide an opinion; (1) Expert Judgement. The distribution of tallies among the bins reflects the expert judgement, based on knowledge of the general ecology of the species and its role on the ecosystem; (2) Limited Data. The data used to distribute the tallies may come from similar species or from other geographic regions out of the Iberian Peninsula; (3) Adequate Data. The score is based on data observed, modelled or directly measured for the species in question and is provided by scientific work carried out in the Iberian Peninsula.

After the individual assessments, a 2-day workshop was carried out where the experts were asked to discuss their evaluations and provide a summarizing text on the likely sign of directional effects of climate change on each species. They were also allowed to modify the distribution of tallies of their votes for the directional effects after the discussion.

#### Regional evaluation

Each expert was asked to perform the evaluation of each indicator independently for each region of Portugal (North, Centre and South; Fig. 1). This procedure made it possible to obtain, for a given species, region-specific assessments of E, S, AC and DE, which could be finally translated into region-specific overall vulnerability assessments.

#### Calculation of the overall vulnerability score

For each species, the number of tallies assigned by the experts to each bin of each indicator was averaged. Then, each tally was assigned a different value in regards of the bin where it was assigned: 1-low, 2-moderate, 3-high, making possible to calculate the value of each indicator by summing the value of the tallies. The final score of the indicator (minimum: 5; maximum: 15) was standardized between 0 and 1. To obtain the value of each dimension of the vulnerability (E, S, or AC) the sum of the values of the related indicators standardized between 0 and 1 was computed. All the indicators had the same weight.

Finally, to calculate the overall vulnerability, the value of each dimension was standardized between 0 and 1, being V calculated as:

2 $$ {\text{V}}_{{{\text{r}} - {\text{cc}}}} = \, \left( {{\text{E}}_{{{\text{r}} - {\text{cc}}}} + {\text{ S}}_{{\text{r}}} } \right) \, {-}{\text{ Ac}}_{{\text{r}}} , $$

where subscripts indicate region (*r)* and climate change (*cc*) specificity, respectively.

The vulnerability score (V_r-cc_) obtained was finally categorized as: “very low vulnerability” (V_r-cc_ < 0.20, note that negative values could exist), low (0.20 < V_r-cc_ < 0.40), moderate (0.40 < V_r-cc_ < 0.60), high (0.60 < V_r-cc_ < 0.80), and very high (V_r-cc_ > 0.80, note that values higher than 1 could exist).

#### Probability of distribution change

The method for assessing the potential for a change in species distribution was adapted from that described in Hare et al.^23^. These authors consider that species with high adult mobility, broadly dispersing early life stages, low habitat specificity, and high temperature sensitivity would have higher potential to change their area of distribution in the context of climate change. Here, we adapted these criteria to the descriptors of vulnerability considered, and calculated the probability of distribution change (*P*) as a function of these indicators considering that high P will be characterized by high adult mobility, long PLD, broadcast egg spawning strategy, wide latitudinal range, and narrow temperature tolerance range. These indicators were standardized between 0 and 1 and then considered as:

3 $$ P = \frac{{\left( {Ad.mobility + PLD + Eggsp.strategy + Lat.range} \right) - temp.range}}{4}. $$

The range of values of *P* was categorized as: very high (*P* > 0.80), high (0.60 < *P* < 0.80), moderate (0.40 < *P* < 0.60), low (0.20 < *P* < 0.40) or very low (*P* < 0.20).

#### Variability in experts’ voting

To evaluate the inter-experts’ variability in the allocation of tallies among the bins of each indicator, a bootstrap analysis was carried out. This analysis consisted of a random sampling (10,000 iterations) with replacement of the total number of tallies allocated per indicator (5 tallies × 3 or 4 experts), calculating the overall vulnerability as described before. Then, the proportion of iterations resulting in a vulnerability of the same category (very low, low, moderate, high, or very high) as the original was computed to estimate the variability in the assignment of vulnerability scores by the experts.

The same procedure was carried out considering the indicators needed to compute the probability of distribution change and the directional effects, allowing to evaluate the certainty on these parameters independently of the overall vulnerability.

#### Vulnerability categories in regards of the relationship between the components of vulnerability

Foden et al.^47^ described four categories of vulnerability based on the relationship between the vulnerability dimensions E, S, and AC. The first category (“highly climate change vulnerable species”) comprises species with high E and S but low AC, which means that they are at great risk due to climate change. The second group (“potential adapter species”) is formed by species with high E, S, and AC, so they may be at risk due to climate change. The third group (“potential persistent species”) considers species with high E and low S and AC, representing those species that may not be at risk due to climate change. Finally, the “high latent risk species” are those with high S and low E and AC, comprising species that would not be currently at risk. Different management perspectives have been proposed for the species within each category (see Foden et al.^47^).

To allocate the species to these categories, we considered “highly climate change vulnerable species” those with very high and high exposure and sensitivity, and low or very low adaptive capacity. Potential adapter species were those with very high, high, or moderate exposure, sensitivity, and adaptive capacity. Potential persistent species were defined as those with very high, high, or moderate exposure and very low or low sensitivity and adaptive capacity. Finally, high latent risk species were considered those with very low or low exposure and adaptive capacity, and very high or high sensitivity.

#### Relationship between overall vulnerability and ecosystem indicators

Aiming at providing a simple but reliable approximation to the assessment of the vulnerability of a given species, we also analysed the relationship between the final vulnerability score and the different indicators used in this work by means of linear regressions.

## Results

### Environmental change

The outputs of the POLCOMS-ERSEM model for the period 2040–2059 under scenarios RCP4.5 and RCP8.5 for SST, pH, salinity, currents, river flow, and zooplankton productivity are shown in the Supplementary SI3 and Table 2. For the SST, a regional increase is expected for the Centre and South of Portugal under both scenarios of climate change, while in the North the average temperature is expected to decrease, overall cooling seems to be caused by offshore rather than coastal waters (Supplementary Fig. SI3-1). The salinity is expected to be lower in the future than in the reference period (Table 2), specially under climate change scenario RCP 8.5 in the three regions, with the drop being higher in a gradient from the North to the South. Ocean acidification is expected to increase (lower pH), especially in coastal waters (Supplementary Fig. SI3-3), with no relevant differences between regions or climate change scenarios. The Eastward currents intensity in the North are expected to increase under both scenarios, especially in RCP 8.5. In the Centre, scenario RCP 4.5 predicts a stronger increase than RCP 8.5. The Northward currents in the South are expected to decrease in intensity under both scenarios (Table 2). The projected plankton concentration is expected to increase under both climate change scenarios in the three regions of Portugal (Table 2). Finally, the river flow is expected to increase under scenario RCP 4.5 and decrease under scenario RCP 8.5.

The weight factors derived from the expected future variability (weight factor 1) are shown in Table 2. These weight factors were standardized between 1 and 2, with the highest variation corresponding to the salinity decrease in the South under climate change scenario RCP 4.5, and the lowest to the river flow variation in the Centre under scenario RCP 8.5.

Following the experts’ criteria, another weight factor (weight factor 2) was defined according to the potential influence of variables on the physiology of organisms. The variable with the highest influence on the physiology of marine organisms was current velocity, due to its relevance for the survival and recruitment of most larval phases. The other variables, ranked in order of importance, were SST, plankton concentration, river flow, salinity and pH. The average between the weight factor 1 and 2 was computed to produce a final weight factor to be included in the vulnerability assessment (Table 2).

### Bootstrap analysis

Bootstrap analyses were carried out to quantify the variability among the experts’ answers (SI6). The bootstrap analysis on the overall vulnerability showed that the assessment of more than 60 species obtained a result equal to the original assessment in more than 80% of the iterations. This indicates a high coherence among the experts’ answers and that the vulnerability assessments are based on uniform criteria. The result of the bootstrap analysis for the probability of distribution change was very similar, with more than 60 species obtaining a result equal to the original assessment in more than 80% of the iterations. The bootstrap analysis on the data quality showed a higher dispersion of the criteria of the experts, with only 25 species achieving a result equal to the original assessment in more than 80% of the iterations.

### Vulnerability assessments

**Figure 2:**
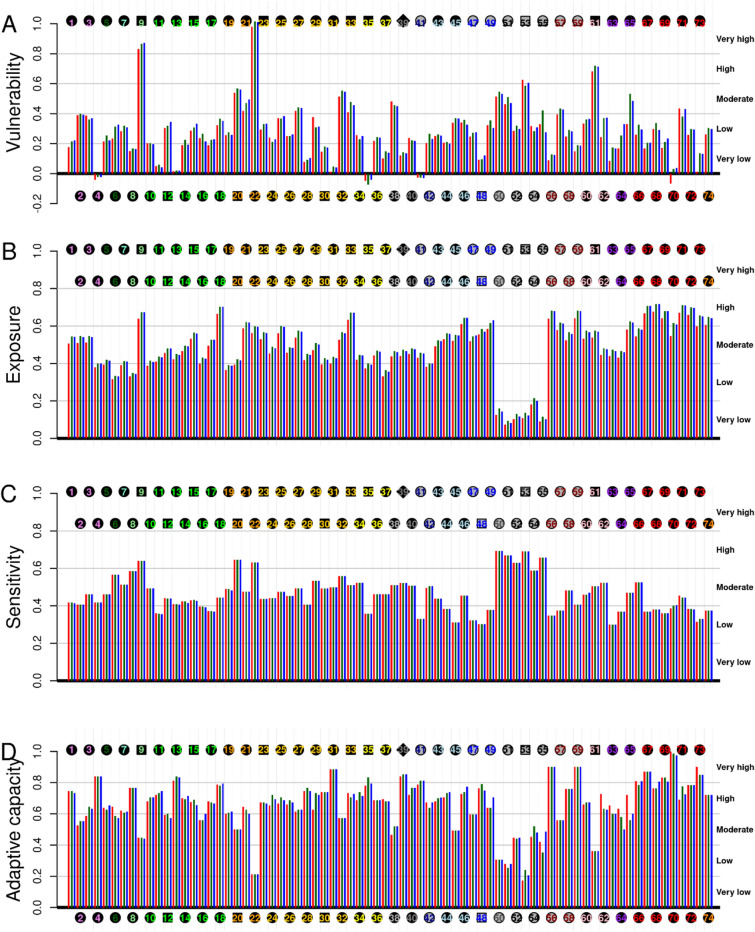
Expert-based assessments of overall vulnerability, exposure, sensitivity, and adaptive capacity for the 74 species under study under climate change scenario RCP 8.5. Species are identified by the species number as in Table 1, and functional groups are represented by the colour of the species number. For each assessment, column colours represent the region (red: North, green: Centre, blue: South). The vulnerability and each of its dimensions are classified in 5 bins (very low, low, moderate, high, and very high). The symbol of the points represents the confidence of the vulnerability assessment obtained during the bootstrap analysis: circle (very high), square (high), diamond (moderate).

The experts’ evaluations for each species are compiled in Supplementary SI4. Vulnerability assessments showed important differences among species. Considering the assessment of the vulnerability under climate change scenario RCP 8.5 (see Supplementary Information 6 for results under climate change scenario RCP 4.5), the vulnerability assessments ranged between *ca.* − 0.05 for species 35 (*Macroramphosus scolopax,* the less vulnerable) and *ca.* + 0.95 for species 22 (*Anguilla anguilla,* the most vulnerable) (Fig. 2A). During the assessment, only *Anguilla anguilla* and species 9 (*Salmo salar*) were ranked as very high, and species 53 and 61 (*Squalus acanthias* -only in the North and South- and *Palinurus elephas*) as high vulnerability. Eleven species were classified as moderately vulnerable (20: *Conger conger*, 21: *Dicentrarchus labrax*, 27: *Umbrina cirrosa*, 32: *Halobatrachus didactylus*, 33: *Cynoscion regalis*, 38: *Scophthalmus maximus*, 50: *Raja clavata*, 51: *Centroscymnus coelolepis*, 55: *Hexanchus griseus—*only in the Centre, 57: *Necora puber—*only in the Centre and South, 65: *Palaemon serratus—*only in the Centre and South, and 71: *Ensis siliqua—*only in the North and South-.

**Figure 3:**
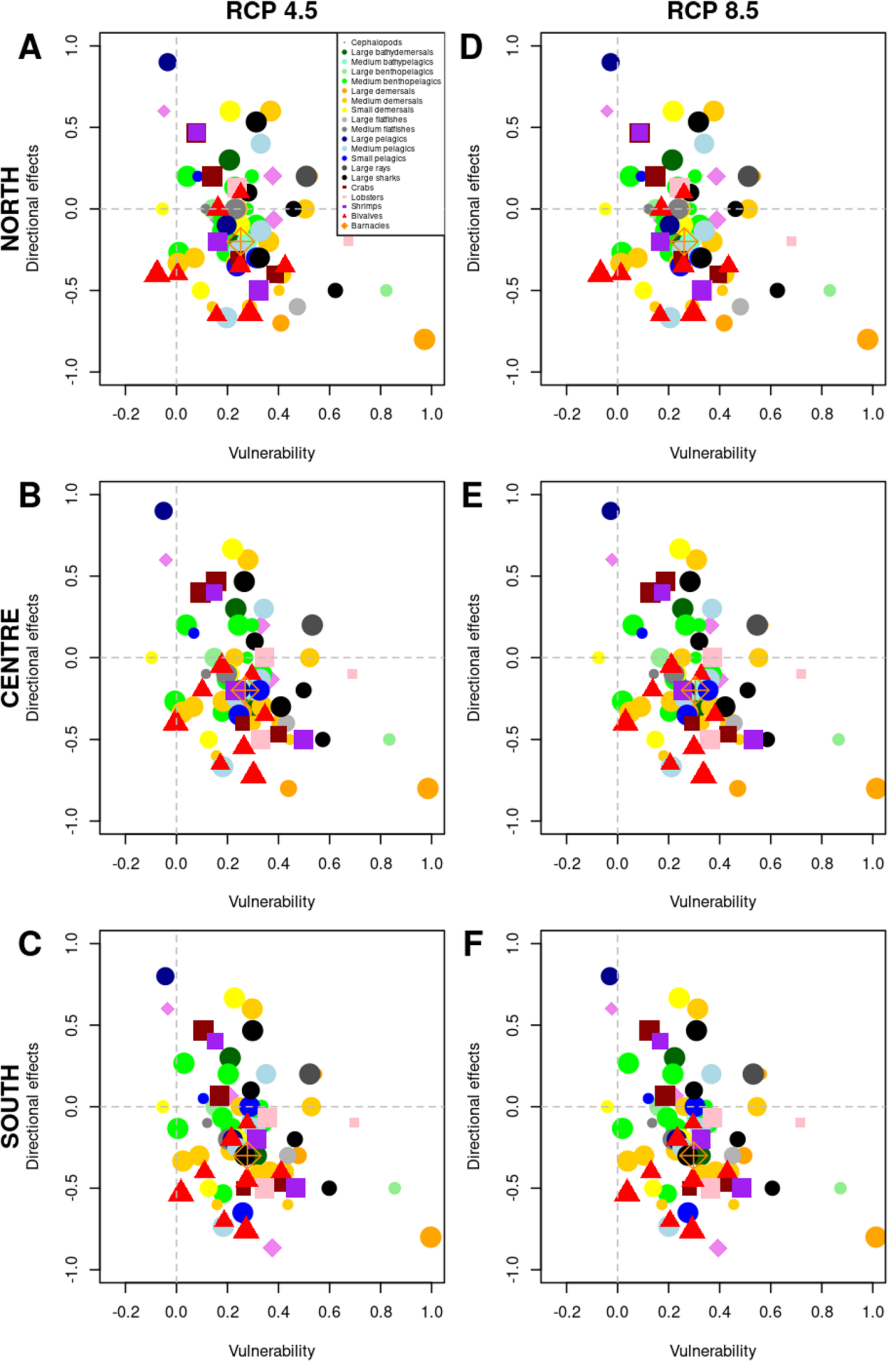
Relationship between overall vulnerability and directional effects for the species under study, considering the three regions of Portugal (North, Centre, and South) and the two scenarios of climate change (RCP 4.5 and RCP 8.5). The size of the points represents the confidence of the vulnerability assessment according to the bootstrap analysis carried out.

The analysis of the vulnerability across regions showed the highest average vulnerability for the Centre region (Fig. 3A–C; Table 3), with no clear pattern on the North–South axis. Considering the scenarios of climate change, the overall vulnerability was found to be higher under climate change scenario RCP 8.5 than RCP 4.5 in the North and Centre, while in the South overall vulnerability was slightly higher under climate change scenario RCP 4.5 (Fig. 3D–F; Table 3). The exposure to environmental change followed the same pattern (Table 3), while the other components of the vulnerability (S, and AC) showed no differences across regions or between climate change scenarios (Table 3). The average estimates of the directional effects were negative in the three regions, with an increasing gradation from the North to the South.

**Table 3:** Average and standard deviation values of overall vulnerability, exposure, sensitivity and adaptive capacity (in a scale of 0–1) and directional effects (in a scale of − 1 to 1) for each region and scenario of climate change based on the 74 species under consideration.

Region	Vulnerability component	RCP 4.5	RCP 8.5
North	Overall vulnerability	0.264 (0.186)	0.272 (0.186)
	Exposure	0.461 (0.136)	0.469 (0.141)
	Sensitivity	0.463 (0.096)	0.463 (0.096)
	Adaptive capacity	0.660 (0.159)	0.660 (0.159)
	Directional effects	− 0.123 (0.357)	
Centre	Overall vulnerability	0.275 (0.186)	0.302 (0.186)
	Exposure	0.475 (0.132)	0.502 (0.146)
	Sensitivity	0.463 (0.096)	0.463 (0.096)
	Adaptive capacity	0.663 (0.159)	0.663 (0.159)
	Directional effects	− 0.136 (0.356)	
South	Overall vulnerability	0.282 (0.184)	0.297 (0.185)
	Exposure	0.482 (0.145)	0.497 (0.148)
	Sensitivity	0.463 (0.096)	0.463 (0.096)
	Adaptive capacity	0.663 (0.157)	0.663 (0.157)
	Directional effects	− 0.164 (0.360)	

No general patterns were found in the overall vulnerability assessments considering the functional groups under study, however, when analysing the components of the vulnerability separately (exposure, sensitivity, and adaptive capacity), the functional groups behaved more as clusters (Fig. 2B–D). Considering the exposure to environmental change, the group of large sharks and rays (species numbers 50–55) showed the lowest levels, while, in general, invertebrates other than cephalopods (species numbers > 55) resulted to be more exposed than cephalopods and fishes (compare the proportion of species classified as high and moderate exposure between both groups of species in Fig. 2B). Considering the sensitivity to environmental change, the results were found to be more or less opposite to the level of exposure, with medium bivalves (species numbers 66–73) showing the higher values (Fig. 2C). Finally, the adaptive capacity was found to be more variable across species. In this case, no significant differences were found between invertebrates and fishes, although the group of large sharks and rays were characterized by low to moderate adaptive capacities. The groups with the less variable inter-specific adaptive capacities were the bivalves (overall very high adaptive capacity), and medium and small demersal fish (species numbers between 23 and 37) with high adaptive capacity.

**Figure 4:**
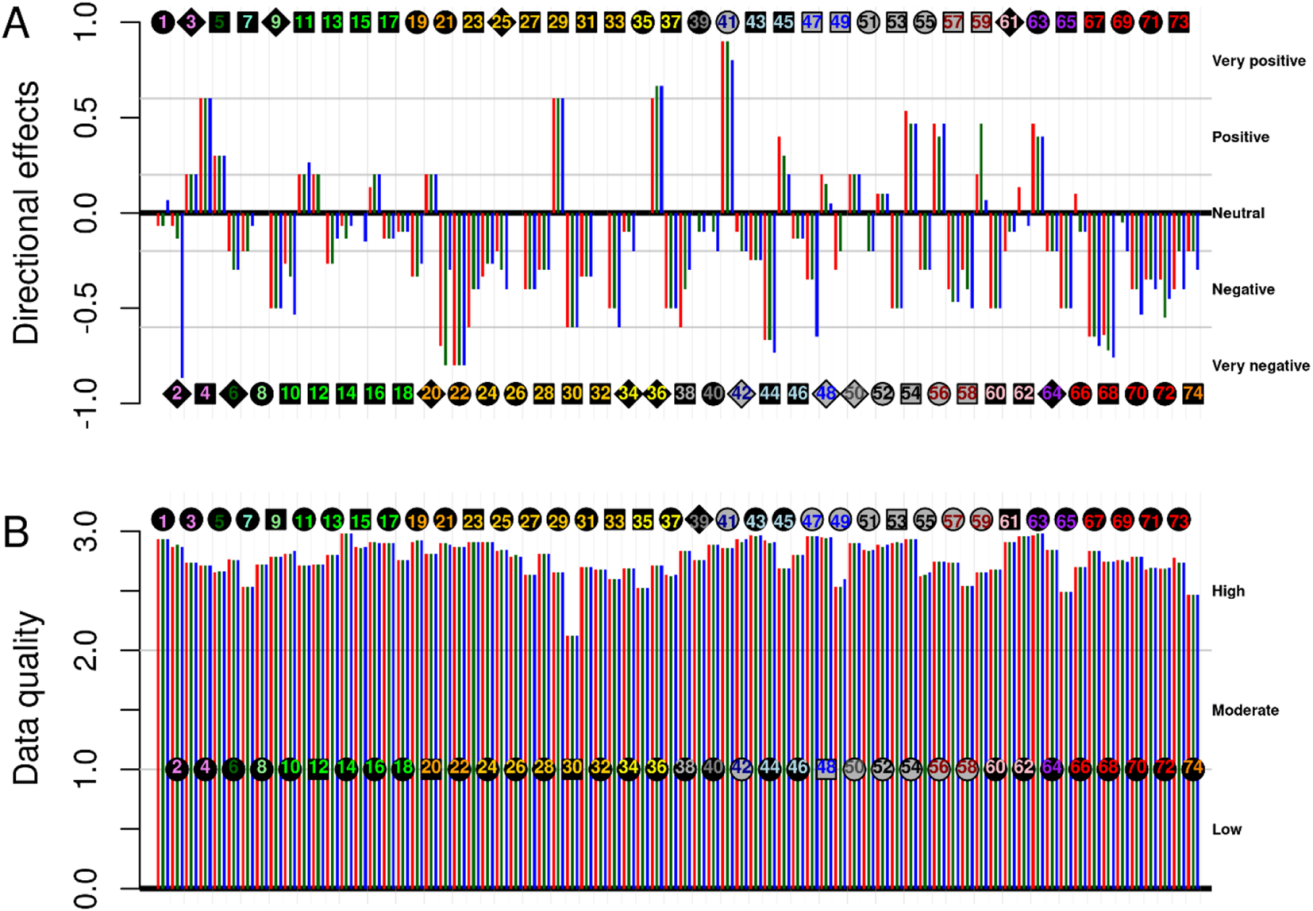
Expert-based assessments of overall directional effects of climate change (**A**), and data quality for the assessment of vulnerability (**B**). Species are identified by the species number, and functional groups are represented by the colour of the species number in the square [see legend in (**A**)]. Geographic regions are represented as in Fig. 2. The symbol of the points in (**A**) represents the confidence in the directional effects’ assessment obtained during the bootstrap analysis: circle (very high), square (high), diamond (moderate). In (**B**), symbols represent the confidence in the bootstrap analysis on the vulnerability assessment. Note that these assessments are common for climate change scenarios RCP 4.5 and RCP 8.5.

The assessment of the directional effects of climate change showed high inter-specific variability, with no clear patterns found among functional groups (except for bivalves, for which overall negative directional effects are expected) (Fig. 4A). In general, of the 74 assessments, 19 were positive in the North, 16 in the Centre, and 18 in the South, while 43 were negative in the North, 47 in the Centre, and 50 in the South. The strength of the directional effects (especially the negative ones) was also evaluated to be higher in the South than in the North and Centre.

The quality of the data used for the assessments was, in general, high (Fig. 4B). A correlation between the quality of the data and the confidence in the vulnerability assessment obtained through the bootstrap analysis was observed (not shown).

**Figure 5:**
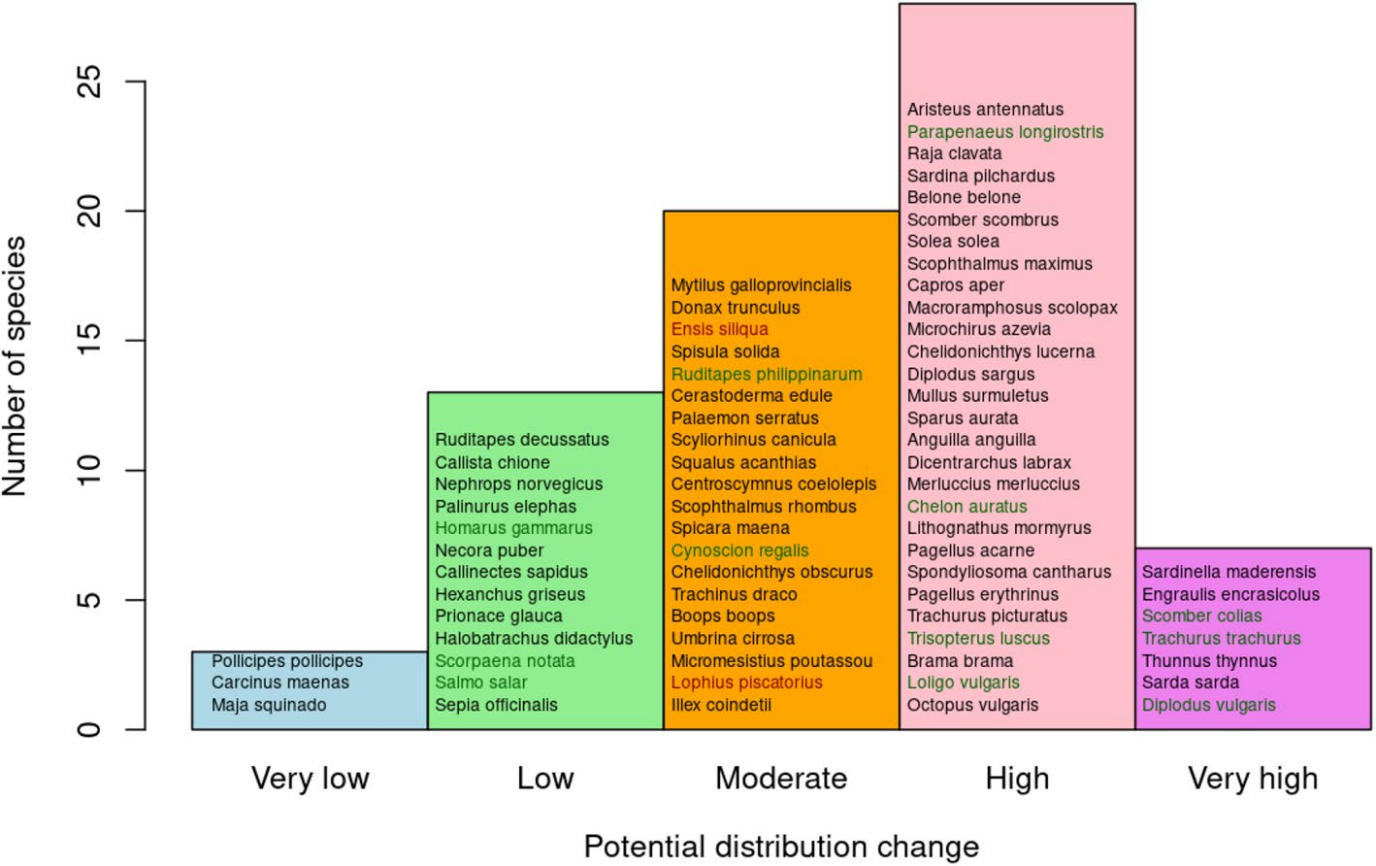
Potential for distribution change. Based on their PLD, adult mobility, egg spawning strategy, latitudinal and temperature tolerance ranges (Eq. 3), the species were ranked for their ability to change their distribution range. Colour fonts represent the confidence in the results for potential distribution change, obtained during the bootstrap analysis: black (very high confidence), green (high confidence), and red (moderate confidence).

The potential for distribution change grouped the species in five categories: very high potential (7 species), high potential (28 species) moderate potential (20 species), low potential (13 species), and very low potential (3 species) (Fig. 5). Six of the seven species with very high potential for distribution change were pelagic fish species, while the three species with very low potential were benthic or sessile crustaceans. The bootstrap analysis showed high coherence among the experts’ votings, with only very high, high and moderate confidences obtained. The species with very high distribution change potential and very high confidence based on the bootstrap analysis were *Sardinella spp.*, *Engraulis encrasicolus*, *Thunnus thynnus*, and *Sarda sarda*.

**Figure 6:**
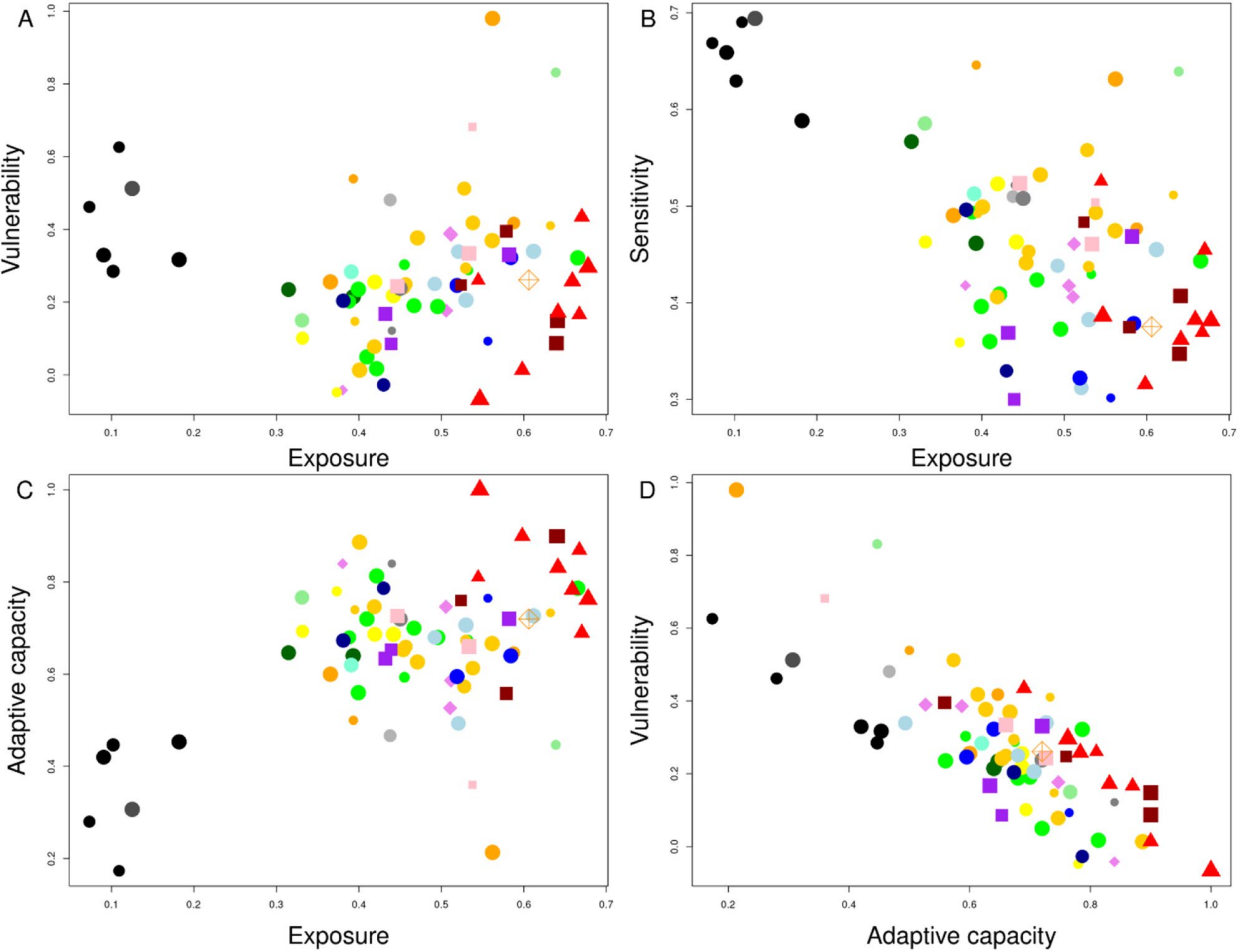
Relationship between species exposure and (**A**) vulnerability, (**B**) sensitivity and (**C**) adaptive capacity for the analysis carried out in the North region under climate change scenario RCP 8.5. Panel (**D**) shows the relationship between the adaptive capacity and the overall vulnerability for the species under study. The size of the points represents the confidence of the vulnerability assessment obtained during the bootstrap analysis. Colour and symbol legend as in Fig. 3A.

To better understand the overall vulnerability scores, the relationship between its different components was evaluated. For each species, the relationship between the level of exposure and vulnerability, sensitivity and adaptive capacity was estimated. The relationship between exposure and overall vulnerability was not significant (p-value = 0.67; Fig. 6A), while the relationship between exposure and sensitivity to environmental change was clearer and more negative (p.value <  < 0.001; Fig. 6B). Similarly, a significant and positive relationship between exposure and the adaptive capacity of species was found (p.value <  < 0.001; Fig. 6C). Finally, the relationship between overall vulnerability and adaptive capacity was strong and negative (p.value < 2 × 10^–16^; r^2^ = 0.796; Fig. 6D).

**Figure 7:**
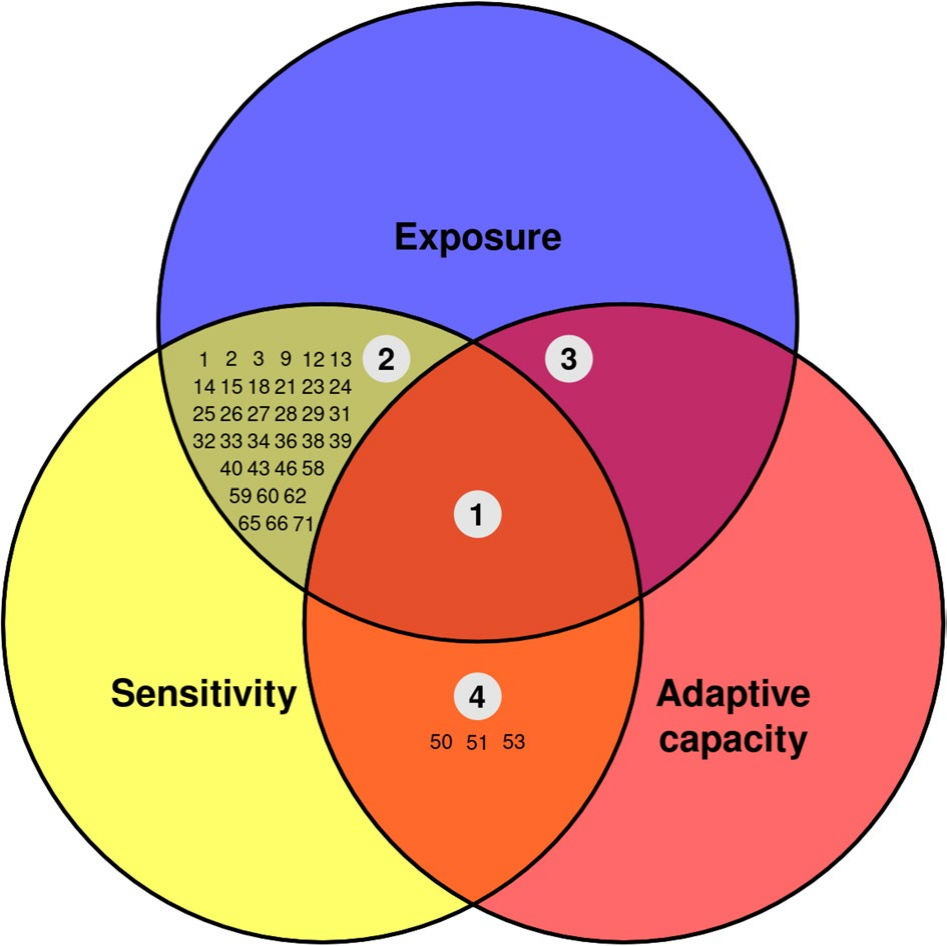
Distribution of species according to the vulnerability categories described by Foden et al.^47^. The combination of the three components of vulnerability define three categories of vulnerability addressing specific conservation implications. Area 1 “highly climate change vulnerable” comprises species with high or very high E and S, and low or very low AC. Area 2 “potential adapters” comprises species with high or very high E, S, and AC. Area 3 “potential persisters ”comprises species with high or very high E, and low or very low S and AC. Area 4 “high latent risk” comprises species with high or very high S, but low or very low E and AC.

The vulnerability categories defined by Foden et al.^47^, based on the relationship between the different components of vulnerability, provide a slightly different perspective of the vulnerability classification of the species. Following this approach, no species was classified as “highly climate change vulnerable” (Fig. 7), but thirty two species were classified as potential adapters to climate change and three as high latent risk: *Raja clavata*, *Centroscymnus coelolepis*, and *Squalus acanthias*.

## Discussion

This is the first attempt to apply an expert-based ecological-trait vulnerability assessment of the effects of climate change on the main marine resources of Portugal. The components of vulnerability (exposure, sensitivity and adaptive capacity) were estimated and discussed by a panel of experts for 74 species, considering three regions of Portugal: North, Centre and South, and two scenarios of climate change: RCP 4.5 and RCP 8.5. This assessment was based on the categorization of a set of biological and ecological traits (“indicators”) of the species into categorical bins. The experts (at least 3 per species) allocated five tallies across the three bins described for each indicator representing their certainty on the voting, hence introducing a gradation of truth in the scoring of the global vulnerability^26^. The panel of experts also evaluated the likely directional effects of climate change across the regions of Portugal, following a similar approach (see Supplementary SI4 for the species-specific evaluation of the indicators of vulnerability and directional effects, as well as the experts’ rationale and bibliography used in their assessment).

The selection of the indicators for the assessment of vulnerability was made with the objective of capturing different life history traits with direct implications on the vulnerability of species to climate change. Hence, for example, while larval dispersive ability could be considered as a single indicator (e.g. Ref.^23^), we split it in different related components aiming to capture a wider variety of life history traits: fecundity, planktonic larval duration, and egg spawning strategy. This procedure is able to captures specific life history traits of interest (e.g., palinurid decapods, which evolved extremely long planktonic larval periods), but also could give rise to correlation among indicators. The issue of the number and correlation of indicators in vulnerability assessments is not new^28,48,49^, but a consensus on the methodology (e.g., number of indicators, treatment of correlations, indicators weighting) has not been reached yet^50^. Something similar happens when considering general *r*/*k* strategies as indicators of vulnerability: while some works consider *r*-strategists as less vulnerable to climate change^23^, it has been demonstrated that, under some circumstances, *r*-strategists could be more affected by climate change^51^. In this study, including some, a priori, correlated indicators in the analysis does not seem to constitute a significant problem because of the way the scores are averaged when calculating the value of the components of vulnerability (E, S, and AC).

A relevant aspect of the present work is the coupling between an expert-based vulnerability assessment and the outputs of a physical-biogeochemical model to weight the exposure of species to the different physical variables^23^. This approach allowed us to infer the vulnerability of the species in regards of two near-future climate change scenarios. Albeit high uncertainty on the evolution of upwelling systems under scenarios of climate change has been acknowledged by the IPCC^52^, the outputs of the model point to an increase in the upwelling pattern characterized by higher intensity of westward currents in the North and Centre, and higher intensity of southward currents in the South coast (Table 2). This could explain the general enhanced productivity of the plankton projected for the three regions and the decrease of average SST in the North. The consequences at the biological scale of enhanced upwelling are yet poorly understood^38,39^, with likely positive effects by the enrichment of surface waters with nutrients from the deep ocean, but also with potential negative effects related to stronger currents that could wash away planktonic larvae, causing massive deaths and negatively affecting recruitment to coastal populations^40^. The effects of increased temperature will affect many aspects of the organisms’ biology and ecology. Higher temperature will shorten biological times^53^ implying that the growth and development rates of organisms will be accelerated, not necessarily at equal velocities^54^. Temperature change would also have consequences for egg size^55^ and mortality rates, in combination with hypoxia issues related to increasing metabolic demand and lowering of dissolved oxygen^56^. Fecundity rates will also be affected as demonstrated by a myriad of works studying the relationship between fish fecundity and temperature in fish and invertebrates^57^. Furthermore, shortening biological times would imply that planktonic larvae and eggs would have less time to disperse, negatively affecting the connectivity between populations. This, however, could be compensated by a higher number of propagules if temperature would enhance the fecundity of females in a complex balance between offspring number and offspring size^41^. Very likely, the final direction of the consequences of increased temperature on the populations of marine organisms would be defined by local conditions (e.g. currents exposure, eutrophication, topographic conditions, etc.)^58,59^ making it difficult to predict global final population dynamics. It is also worth to mention that this study does not consider non-climate stressors that could be already affecting the sensitivity and adaptive capacity of marine organisms (e.g., pollution, relevant land use change, marine traffic and noise, appearance of invasive species, changing demand by consumers, etc.)^60^.

The two climate change scenarios considered in this work assume different pathways of CO_2_ emissions along the XXI century to predict global warming increase of 1.8 °C (RCP 4.5) or 3.7 °C (RCP 8.5). To the date, historical total cumulative CO_2_ emissions are in closer agreement with scenario RCP 8.5, making it the most plausible near to midterm scenario of global warming^61^. In this scenario, overall environmental variability is expected to be higher than under RCP 4.5 (Table 2), which in our vulnerability assessment translates into higher exposures to climate change for marine organisms. A strong correlation between vulnerability and adaptive capacity was found under both climate change scenarios (Fig. 6D and see Supplementary SI6 for RCP 4.5 results), which is, probably, a consequence of the nature of the organisms inhabiting the Portuguese marine ecosystem. In temperate ecosystems, species are subject to high annual environmental variability in relation to tropical or subtropical ecosystems were conditions are more constant. This causes organisms from temperate systems to show high plasticity and adaptive capacity, which also explains their overall low vulnerability to climate change^62^.

Of the 74 species analysed under scenario RCP 8.5, only two (*Anguilla anguilla* and *Salmo salar*) were ranked as very high vulnerability, two (*Squalus acanthias* and *Palinurus elephas*) as high, and eleven as moderately vulnerable. In scenario RCP 4.5, results were slightly different and only *Anguilla anguilla* was ranked as very high, *Salmo salar* as high, and five species as moderate (Supplementary SI6). High variability in the overall vulnerability scores was found within the different functional groups, although they behaved more as clusters when evaluating the different components of the vulnerability (exposure, sensitivity, and adaptive capacity). On a regional basis, the results from our analysis showed the highest ecological vulnerabilities in the Centre region, with no clear North–South pattern. This variation was mostly driven by regional differences in the level of exposure to climate change, as the other components of the vulnerability (sensitivity and adaptive capacity) were mostly the same among regions (Table 3). This is because the available information on the ecology of the species does not allow to allocate the tallies differentially among the bins of the indicators of sensitivity and adaptive capacity when considering the different regions, while the level of exposure depends on the outputs of the physical-biogeochemical model, which are region-specific.

We also found that the relationship between the overall vulnerability and the adaptive capacity of the species considered in this study was very strong (Fig. 6D; r^2^ = 0.796) as a result of the negative (but weaker) relationship between exposure and sensitivity (Fig. 6B). In this way, the adaptive capacity here proposed could be seen as a proxy for the overall vulnerability of the species based on five criteria. Similarly, aiming at providing a simple but reliable approximation to the assessment of the vulnerability of the species, we analysed the relationship between the overall vulnerability score and the different indicators used in this work (Supplementary Fig. S7-1). In general, no single indicator was found to be a good proxy per se, but the ones with the highest relationship were the ones related to the adaptive capacity of the species: the ICES stock status, the replenishment potential, the IUCN vulnerability status, and, to a lesser extent, the vulnerability to fisheries as described by Cheung et al.^26^. The same exercise was carried out for the directional effects, although in this case no clear relationship was found (Supplementary Fig. SI7-2). This result was not surprising, however, since the directional effects of climate change resulted to be highly variable among the species of a given functional group, depending on the interaction of many different biological, ecological and exploitation factors.

Based on the contribution of specific indicators, the distribution change probability of the species was also estimated. This metric is of special interest to fisher communities by identifying the resources with higher potential for climate-driven distribution shifts. The displacement of species to higher latitudes due to warming poses a challenge for small scale fisheries, as it would require adaptations that could be expensive or technologically complex for the fleet in its current state^63^. In our study, the species identified as very high potential for distribution change are of great economic relevance (Fig. 5) and some of them are captured by the coastal fleet (i.e. *Trachurus trachurus*, *Diplodus vulgaris*, *Scomber colias*), whose traditional character would probably impede costly adaptations to make longer displacements for fish, questioning the future of this sector in its current form.

Assessing the vulnerability of the main commercial species of fish and invertebrates establishes the basis for the assessment of the vulnerability of the main fisheries (or any other activity relying directly or indirectly on these resources) of Portugal^36^. The connection between the biological vulnerability of target species and the vulnerability of human societies has been linked in different ways in the literature. Hence, some works estimate the vulnerability of societies based on the evaluation of social indicators, independently of the biological vulnerability of target species (e.g. Refs.^32,64^), while others included the biological vulnerability as a component of the vulnerability of societies (e.g. Refs.^33,34^). A first attempt to estimate the vulnerability of the main marine fisheries of Portugal was carried out by Gamito et al.^65^. The methodology relied on social indicators obtained by means of social statistics and interviews with fishermen to assess their exposure and sensitivity. Despite the IPCC approach not being followed, similar overall vulnerability results to the ones described here were obtained at the regional scale, with the South achieving higher vulnerability levels than the Centre and North. For the fisheries evaluated, the highest vulnerability obtained was for purse-seiners due to the high climate change vulnerability of sardine and because of a low capacity to adapt their gears or target species.

Vulnerability assessments based on expert elicitation have been criticized because they can wrongly assess the vulnerability of migratory species^66^. Arguments in this regard indicate that vulnerability assessments do not properly consider the species’ migratory status, or that ecological traits measured at non-breeding grounds are usually considered to describe the biology of the species. In addition, the moment of migration is rarely considered in these assessments as it requires very specific physiological changes or implies specific dangers for the survival of individuals. Hence, because these species spend only one part of their life cycle within the region of study, full life-cycle assessments should be preferentially used. The present work deals with this issue by incorporating a “seasonal migrations” sensitivity indicator and several others related to the egg or larval phase (PLD, egg spawning strategy, duration of spawning period, and larval habitat during the exposure assessment). In our results, the long-distance migratory species (i.e. *Anguilla anguilla*, *Salmo salar*, *Aphanopus carbo*, *Thunnus thynnus*), were classified high or very high in the vulnerability ranking. Similarly, despite the general perception that deep ocean biodiversity is less exposed to climate change, it has been recently found that these organisms might be more vulnerable to climate change because the climate velocity of deep waters (km year^−1^^67^) is faster than surface climate velocity^68^. In this regard, the present work succeeded in capturing the vulnerability of deep-water organisms (e.g. *Centroscymnus coelolepis*, species 51) by considering aspects of their biology and ecology through the indicators of sensitivity and adaptive capacity, but probably failed at capturing their real exposure to climate change, very likely higher than estimated.

The categories of vulnerability defined by Foden et al.^47^ are accompanied by implications for conservation, prioritisation, and strategic management. According to this classification, “highly vulnerable” species would require specific research and intervention to warrant survival. No species was classified in this category in the present study, although the European eel (*A. anguilla*) achieved the highest exposure and sensitivity, and the lowest adaptive capacity over the three regions and climate change scenarios. The European eel is classified as “critically endangered” by the IUCN and has specific action recovery plans and systematic monitoring schemes at European level. The category of “potential adapters” to climate change described by Foden et al.^47^ also would require monitoring and supporting adaptive responses. Thirty-four species were identified within this category, most of them classified by the IUCN as least concern and only one as vulnerable in European waters (*Salmo salar*). The vulnerability assessment also highlighted the group of sharks and rays, with a high proportion of species over the average vulnerability of the species under study. This group possess specific life-history traits such as low fecundity and direct development making them especially vulnerable to fishing, lowering their adaptive capacity in the context of climate change (Gallagher et al., 2012)^69^.

The outcome of this work is the identification of the most vulnerable marine commercial fish and invertebrates of Portugal. The assessment was carried out over three regions of Portugal and two different scenarios of climate change, and the results identified some long-migratory species within the most vulnerable, in concordance with the overall European and national protection levels already established. Higher vulnerabilities were found in the Centre of Portugal than in the North and the South, due to higher expected environmental variability. Among climate change scenarios, RCP 8.5 vulnerabilities were higher than RCP 4.5 vulnerabilities. This work also states the basis for the assessment of the climate change vulnerability of activities relying on marine resources in Portugal.

## Supplementary Information

Supplementary Information 1.

Supplementary Information 2.

Supplementary Information 3.

Supplementary Information 4.

Supplementary Information 5.

Supplementary Information 6.

Supplementary Information 7.
