# Supplementary material for: Climate change vulnerability assessment of the main marine commercial fish and invertebrates of Portugal
Source: Sci Rep. 2021 Feb 3;11:2958. doi: 10.1038/s41598-021-82595-5 (PMC7858592; doi:10.1038/s41598-021-82595-5)
Supplement: Supplementary file 1 — Supplementary Information 1. [file 41598_2021_82595_MOESM1_ESM.pdf]

Climate change vulnerability assessment of the main marine commercial fish and invertebrates of Portugal

SUPPLEMENTARY INFORMATION 1:

Selection of species

Juan Bueno-Pardo[1*], Daniela Nobre[1], João N. Monteiro[1], Pedro M. Sousa[1], Eudriano F. S. Costa[1], Vânia Baptista[1], Andreia Ovelheiro[1], Vasco M. N. C. S. Vieira[2], Luís Chícharo[3], Miguel Gaspar[4], Karim Erzini[1], Susan Kay[5], Henrique Queiroga[6], Maria A. Teodósio[1], Francisco Leitão[1]

[1] Centro de Ciências do Mar (CCMAR), Universidade do Algarve, Campus de Gambelas, Faro 8005-139, Portugal

[2] Instituto Superior Técnico, Lisboa 1041-001, Portugal

[3] Faculdade de Ciência e Tecnologia, Universidade do Algarve, Campus de Gambelas, Faro 8005-139, Portugal

[4] Instituto Português do Mar e a Atmósfera (IPMA), Centro de Olhão, Olhão 8700-305, Portugal

[5] Plymouth Marine Laboratory, Prospect Place, The Hoe, Plymouth PL1 3DH, UK

[6] Departamento de Biologia e Centro de Estudos do Ambiente e do Mar (CESAM), Universidade de Aveiro, Campus Universitário de Santiago, Aveiro 3810-193, Portugal

* Corresponding author: jbuenopardo@gmail.com

Table SI1-1. Selection of species according to the landings criterion.

Official landings data from the Direção Geral de Recursos Marinhos (DGRM) were gathered between 1989 and 2015 in continental Portugal. These data were reported by annual landings (kg) per port. Ports were then assigned to each region of Portugal according to Fig. 1 in the main text. Three gears were reported: purse-seine (small scale fisheries comprising a wide variety of techniques), trawling (comprising both bottom and pelagic trawling), and purse-seine. For each combination of region and gear we considered the most landed species accounting for 70% along the time-series.

**Region**	**Gear**	**Species accounting for 70% of landings**
North	Purse-seine	*Sardina pilchardus*
	Trawling	*Trachurus trachurus, Micromesistius poutassou, Trisopterus luscus, Scomber colias, Trachurus picturatus*
	Multigear	*Sardina pilchardus, Trisopterus luscus, Trachurus trachurus, Octopus spp., Octopus vulgaris, Cerastoderma edule, Ruditapes decussatus, Merluccius merluccius, Conger conger, Scomber japonicus, Spisula solida*
Centre	Purse-seine	*Sardina pilchardus*
	Trawling	*Trachurus trachurus, Micromesistius poutassou, Merluccius merluccius, Trachurus picturatus, Parapenaeus longirostris*
	Multigear	*Aphanopus carbo, Scomber japonicus, Octopus spp., Sardina pilchardus, Lepidopus caudatus, Trachurus trachurus, Octopus vulgaris, Sepia officinalis, Centroscymnus coelolepis, Pharus legumen, Conger conger, Merluccius merluccius*
South	Purse-seine	*Sardina pilchardus*
	Trawling	*Parapenaeus longirostris, Trachurus trachurus, Micromesistius poutassou, Merluccius merluccius, Octopus spp., Lophius spp., Sepia officinalis, Aristeus antennatus*
	Multigear	*Octopus spp., Scomber japonicus, Sardina pilchardus, Octopus vulgaris, Spisula solida, Trachurus trachurus, Sepia officinalis, Merluccius merluccius, Donax spp., Conger conger*

Table SI1-2. Selection of species according to total revenue between 1989 and 2015 in continental Portugal.

Data on first market price per species (€/kg) was available from the DGRM between 1989 and 2015. Considering this price and the total landings per species (kg) we calculated the average revenue of the species during the time series (€). We finally considered the species accounting for more than 3% of the total revenue per combination of region and gear.

Region	Gear	Species representing more than 3% of total revenue
North	Purse-seine	*Sardina pilchardus, Scomber japonicus*
	Trawling	*Trachurus trachurus, Micromesistius poutassou, Trisopterus luscus, Scomber scombrus, Trachurus picturatus*
	Multigear	*Dicentrarchus labrax, Psetta maxima, Scophthalmus rhombus, Solea spp., Dicentrarchus spp., Anguilla anguilla*
Centre	Purse-seine	*Sardina pilchardus, Scomber japonicus*
	Trawling	*Trachurus trachurus, Micromesistius poutassou, Merluccius merluccius, Trachurus picturatus, Parapenaeus longirostris*
	Multigear	*Dicentrarchus spp., Sparus aurata, Octopus spp., Dicentrarchus labrax, Solea solea, Psetta maxima, Sepia officinalis, Solea spp.*
South	Purse-seine	*Sardina pilchardus, Scomber japonicus, Osteichthyes*
	Trawling	*Parapenaeus longirostris, Trachurus trachurus, Micromesistius poutassou, Merluccius merluccius, Octopus spp., Lophius spp., Sepia officinalis, Aristeus antennatus*
	Multigear	*Octopus spp., Scomber japonicus, Sardina pilchardus, Octopus vulgaris, Spisula solida, Trachurus trachurus, Sepia officinalis, Merluccius merluccius, Donax spp., Conger conger*

Table SI1-3. Species considered as important in the discards.

For the three *métiers* considered by the DGRM, we obtained information on the most discarded species from the work of Leitão et al. (2014). The top-ten species discarded by *métier* were considered here.

**Métier**	**Top-ten discarded species**
Purse-seine	*Boops boops, Scomber colias, Belone belone, Sarina pilchardus, Macroramphosus scolopax, Scomber scombrus, Halobatrachus didactylus, Spicara flexuosa, Trachurus trachurus*
Trawling	*Trachurus picturatus, Merluccius merluccius, Scomber colias, Micromesistius poutassou, Trachurus trachurus, Capros aper, Chondrichthyes, Boops boops, Conger conger, Sardina pilchardus, others*
Multigear	*Liza aurata, Scomber colias, Sardina pilchardus, Trachinus draco, Boops boops, Microchirus azevia, Chelidonichthys obscurus, Merluccius merluccius, Scorpaena notata, Pagellus acarne*

Table SI1-4. Can industry species.

A telephonic and e-mail search was performed considering the most important can entreprises of Portugal. Some species were only specified to the genus level. In these cases we did not add new species to the final list.

**Enterprise**	**Species**
Portal das Conservas	*Thunnus spp., Gadus morhua, Trachurus trachurus, Scomber colias, Anguilla anguilla, Merluccius merluccius, Belone belone, Salmo salar, Sardina pilchardus*
Conservas Ramirez	*Sardina pilchardus, Thunnus spp., Scomber colias, Trachurus trachurus, Gadus morhua, Ilex coindetti, Loligo spp., Mytilus galloprovincialis*
Conserveira do Sul	*Sardina pilchardus, Thunnus spp., Aristeus antennatus, Scomber colias, Trachurus trachurus, Brama brama, Engraulis encrasicolus,*
Dâmaso	*Thunnus spp.*
Poveira	*Sardina pilchardus, Scomber colias, Thunnus spp., Gadus morhua, Salmo salar*
Ala Arriba	*Thunnus spp.*
Alva	*Thunnus spp., Sardina pilchardus, Scomber colias, Gadus morhua*
Capitão Poveiro	*Thunnus spp., Sardina pilchardus*
Galeão	*Thunnus spp., Scomber colias, Trachurus trachurus*
Lapa	*Thunnus spp., Sardina pilchardus, Scomber colias, Gadus morhua*
Minerva	*Thunnus spp., Gadus morhua, Sardina pilchardus, Scomber colias*
Taby	*Thunnus spp., Sardina pilchardus, Engraulis encrasicolus*
La Gondola	*Sardina pilchardus, Scomber colias, Octopus spp., Thunnus spp., Gadus morhua, Trachurus trachurus, Aphanopus carbo, Merluccius merluccius, Loligo spp., Salmo trutta, Salmo salar, Engraulis encrasicolus, Petromyzon marinus*
Conservas Nero	*Aphanopus carbo*
Açor	*Thunnus spp., Gadus morhua, Salmo salar*
Catraio	*Thunnus spp.*
Naval	*Thunnus spp., Gadus morhua, Trachurus trachurus*
Georgette	*Sardina pilchardus, Engraulis encrasicolus, Scomber colias, Salmo salar*
Pinhais & Cia.	*Sarina pilchardus, Scomber colias, Trachurus trachurus*
Cofisa	*Thunnus spp., Sardina pilchardus, Scomber colias, Octopus spp., Ilex coindetti, Gadus morhua, Trachurus trachurus, Cerastoderma edule, Mytilus edulis*
Cofaco	*Thunnus spp., Sardina pilchardus, Gadus morhua, Loligo spp., Mytilus galloprovincialis, Ilex coindetti, Scomber colias, Engraulis encrasicolus*

Table SI1-5. Most landed species in Morocco.

The department of sea fisheries of the Kingdom of Morocco (Departement de la pêche maritime, 2017) reported the most landed species in Moroccan waters in 2017. Due to the potential northward displacement of some of these species caused by global warming, we considered these species in our study as eventual new targets for Portuguese fisheries. In some cases, the species were reported uniquely by their French common name, so an assignment to their scientific name was made:

French name reported	Scientific name	Source
Sardine	*Sardine pilchardus*	Departement de la Pêche Maritime, 2017
Maquereau	*Scomber colias*	Departement de la Pêche Maritime, 2017
Anchois	*Engraulis encrasicolus*	Departement de la Pêche Maritime, 2017
Chinchard	*Trachurus trachurus*	Departement de la Pêche Maritime, 2017
Sardinelle	*Sardinella spp.*	Departement de la Pêche Maritime, 2017
Thonidés	*Thunnus spp.*	Departement de la Pêche Maritime, 2017
Loup	*Dicentrarchus labrax*	Departement de la Pêche Maritime, 2017
Dorade	*Sparus aurata*	Departement de la Pêche Maritime, 2017
Grondin	*Chelidonichthys lucerna*	Departement de la Pêche Maritime, 2017
Merlu	*Merluccius merluccius*	Departement de la Pêche Maritime, 2017
Ombrine	*Umbrina cirrosa*	Departement de la Pêche Maritime, 2017
Pageot	*Pagellus erythrinus*	Departement de la Pêche Maritime, 2017
Sole	*Solea solea*	Departement de la Pêche Maritime, 2017
NA	*Sardina pilchardus*	FAO (2011-2019)
NA	*Scomber colias*	FAO (2011-2019)
NA	*Osteichthyes*	FAO (2011-2019)
NA	*Octopodidae*	FAO (2011-2019)
NA	*Trachurus spp.*	FAO (2011-2019)
NA	*Engraulis encrasicolus*	FAO (2011-2019)
NA	*Sepiidae*	FAO (2011-2019)
NA	*Rhodophyceae*	FAO (2011-2019)
NA	*Sardinella spp.*	FAO (2011-2019)
NA	*Loliginidae*	FAO (2011-2019)
NA	*Natantia*	FAO (2011-2019)
NA	*Merluccius merluccius*	FAO (2011-2019)
NA	*Sparidae*	FAO (2011-2019)
NA	*Boops boops*	FAO (2011-2019)
NA	*Pleuronectiformes*	FAO (2011-2019)
NA	*Thunnus thynnus*	FAO (2011-2019)
NA	*Trichiurus lepturus*	FAO (2011-2019)

NA	*Cyprinidae*	FAO (2011-2019)
NA	*Sarda sarda*	FAO (2011-2019)
NA	*Triglidae*	FAO (2011-2019)

Bibliography

- FAO 2011-2020. Fisheries and aquaculture software. FishStatJ - Software for Fishery and Aquaculture Statistical Time Series. In: FAO Fisheries Division [online]. Rome. Updated 14 September 2020. [Cited 24 November 2020]. http://www.fao.org/fishery/

- Département de la Pêche Maritime (2017) Mer en chiffres. Royaume du Maroc. Ministère de l’Agriculture, de la Pêche Maritime, du Développement Rural et des Eaux et Forêts.

- Leitão, F.; Baptista, V.; Zeller, D.; Karim, E. (2014) Reconstructed catches and trends for mainland Portugal fisheries between 1938 and 2009: implications for sustainability, domestic fish supply and imports. Fisheries Research 155: 33-50.
