# Supplementary material for: Climate change vulnerability assessment of the main marine commercial fish and invertebrates of Portugal
Source: Sci Rep. 2021 Feb 3;11:2958. doi: 10.1038/s41598-021-82595-5 (PMC7858592; doi:10.1038/s41598-021-82595-5)
Supplement: Supplementary file 2 — Supplementary Information 2. [file 41598_2021_82595_MOESM2_ESM.pdf]

Climate change vulnerability assessment of the main marine commercial fish and invertebrates of Portugal

SUPPLEMENTARY INFORMATION 2:

Description of the indicators used during the vulnerability assessment

Juan Bueno-Pardo[1*], Daniela Nobre[1], João N. Monteiro[1], Pedro M. Sousa[1], Eudriano F. S. Costa[1], Vânia Baptista[1], Andreia Ovelheiro[1], Vasco M. N. C. S. Vieira[2], Luís Chícharo[3], Miguel Gaspar[4], Karim Erzini[1], Susan Kay[5], Henrique Queiroga[6], Maria A. Teodósio[1], Francisco Leitão[1]

[1] Centro de Ciências do Mar (CCMAR), Universidade do Algarve, Campus de Gambelas, Faro 8005-139, Portugal

[2] Insitituto Superior Técnico, Lisboa, Portugal

[3] Faculdade de Ciência e Teconologia, Universidade do Algarve, Campus de Gambelas, Faro 8005-139, Portugal

[4] Instituto Português do Mar e a Atmósfera (IPMA), Centro de Olhão, Olhão 8700-305, Portugal

[5] Plymouth Marine Laboratory, Prospect Place, The Hoe, Plymouth PL1 3DH, UK

[6] Departamento de Biologia e Centro de Estudos do Ambiente e do Mar (CESAM), Universidade de Aveiro, Campus Universitário de Santiago, Aveiro 3810-193, Portugal

* Corresponding author: jbuenopardo@gmail.com

TABLE OF CONTENTS

Exposure factors related to climate change. ... 3
1. Sea surface temperature (SST). ... 3
2. Currents. ... 3
3. pH. ... 4
4. Salinity. ... 4
5. Extreme events. ... 5
6. Riverflow / rainfall. ... 5
7. Plankton productivity. ... 6
Attributes related to the species' sensitivity to climate change. ... 7
8. Trophic level. ... 7
9. Fecundity. ... 7
10. Semelparity/Iteropary. ... 8
Semelparous species die short after reproductive events, while iteroparous species pass through several reproductive events during their lifespan. Due to their lower reproductive output, semelparous species are more sensitive to climate change than iteroparous species. ... 8
11. Egg spawning strategy. ... 8
12. Von Bertalanffy K. ... 8
13. Age at maturity. ... 9
14. Population growth rate (r). ... 9
15. Longevity. ... 9
16. Gonochorism. ... 10
17. Spawning cycle ... 10
18. Planktonic larval duration (PLD). ... 10
19. Latitudinal range. ... 11
20. Temperature range. ... 11
21. Adult mobility. ... 12
22. Seasonal migrations or reproductive movements. ... 12
23. Species sociability. ... 12
24. Complexity of reproductive strategy. ... 13
Attributes related to the species' adaptive capacity to climate change. ... 14
25. ICES stock status. ... 14
26. Replenishment potential. ... 14
27. Vulnerability according to the International Union for Conservation of Nature (IUCN). .. 15
28. Vulnerability to fisheries. ... 15
29. Fishing pressure ... 16
Directional effects of climate change. ... 17
30. Predicted directional effect of climate change. ... 17
Data quality. ... 19
Bibliography. ... 20

Exposure factors related to climate change.

Exposure is defined as the predicted environmental change that the stock may experience within its range of occurrence. It represents the overlap between the stock's distribution and the magnitude of a spatial distribution of the expected environmental change.

1. Sea surface temperature (SST).

According to the POLCOMS-ERSEM simulations (Fig. SI2-1, and Table 1 in the main text), average SST is expected to increase in the South and Centre regions for the period 2040-2059 in relation to 2000-2019, under scenarios RCP4.5 and RCP8.5. In the North, however, average SST is expected to decrease during the same period. The average change could attain +0.5ºC in the South, while in the North cooling could be of around -0.2ºC. Changes are expected to be more drastic under scenario RCP8.5 (Fig. SI2-1B). Cooling in the North could be caused by an increase of upwelling events, where they are stronger and more frequent (Leitão et al., 2019). Temperature has direct consequences on the developmental time of organisms, their fecundity, and their size (Bueno and López-Urrutia, 2012; Gillooly et al., 2002), affecting both the adult and larval/young phases.

Exposure to changes in SST is measured based on the proportion of time that a given species spends in surface waters (<25 m, where temperature changes occur faster) and the habitat occupied by the dispersive larvae.

- Low exposure: adults spend less than 50% of time in surface waters and larvae are not planktonic.
- Moderate exposure: adults spend less than 50% of the time in surface waters and larvae are planktonic.
- High exposure: adults spend more than 50% of the time in surface waters and larvae are planktonic.

Examples: *Centroscymnus coelolepis* occurs between 128 and 3700 m and their development is direct with no planktonic larvae, so exposure should be classified as low. *Aphanopus carbo* occurs at depths between 200 and 2300 m and their larvae are planktonic so should be classified as moderate exposure. *Carcinus maenas* spends all its life in surface waters and their larvae are planktonic, so should be classified as high exposure.

2. Currents.

Westward currents in the North and Centre, and Southward currents in the South can be interpreted as a proxy for upwelling index in Portuguese waters. An intensification of the upwelling index in the South has been identified in recent decades, while in the North and Central regions no trend has been found (Leitão et al., 2019). Nevertheless, westerly and northerly currents are expected to increase for the decade of 2040-2050 in the context of climate change (Bakun, Dubert, refs). The POLCOMS-ERSEM model also predicts an increase of westward and southward currents (Fig. SI2-2 and Table 1 in the main text).

The consequences of enhanced upwelling in the Portuguese coast are difficult predict, due to the interaction of different variables. While deep nutrient-rich waters are expected to reach surface layers more oftenly increasing photosynthesis and primary productivity, stronger and more frequent upwelling events could also imply enhanced offshore surface currents that could wash out planktonic larvae, affecting negatively the recruitment to coastal habitats (Bakun et al., 2015). For these reasons, species with planktonic stages are more exposed to changes in currents than species with no planktonic larvae.

- Low exposure: benthic or demersal species with no planktonic stage of development.
- Moderate exposure: benthic or demersal species with planktonic stage of development.
- High exposure: pelagic species with planktonic stage of development.

<u>Examples:</u> *Prionace glauca* development does not imply a planktonic larva, so should be classified as low exposure. *Sepia officinalis* inhabits coastal areas and has a larval phase with active swimming behaviour, so it should be classified as moderate exposure. *Necora puber* has planktonic larvae with relatively long planktonic duration and moderate swimming ability, so should be classified as high exposure.

3. pH.

pH is expected to decrease due to the emission of greenhouse gases to the atmosphere and the dissolution of CO_2 into seawater. Similar changes are expected for scenarios RCP4.5 and RCP8.5 between 2040 and 2059 in relation to 2000-2019, with a decrease of around 0.02 units (Fig. SI2-3, Table 1 of the main text). As the acidification is expected to be more pronounced in surface layers (Fig. SI2-3), open ocean organisms would be less exposed to pH change than inshore or estuarine species. Additionally, organisms with calcareous structures that need to precipitate calcium carbonate to growth are also more exposed than non-calcareous organisms.

- Low exposure: open ocean / deep water species, or organisms without calcareous structures.
- Moderate exposure: continental shelf species, or organisms feeding on calcareous organisms.
- High exposure: inshore / coastal species, or organisms with external calcareous structures.

<u>Examples:</u> *Aphanopus carbo* is a species inhabiting deep waters, so should be classified as low exposure. *Diplodus sargus* is a continental shelf species feeding on small crustaceans, so should be classified as moderate exposure. *Cerastoderma edule* is a calcareous organism inhabiting estuaries, so should be classified as high exposure.

4. Salinity.

Salinity is expected to decrease between 2040 and 2059, especially in coastal waters (Fig. SI5). This decrease is expected to be more pronounced under scenario RCP 8.5 than in RC P4.5 due to enhanced continental ice melt and consequent sea level rise.

Salinity variations could affect the osmoregulation of organisms, affecting vital processes (Kinne, 1966). Nevertheless, salinity effects are largely underrepresented in climate change impact assessments. Estuarine/inshore organisms are more exposed to variations in salinity than open ocean or deep water species.

- Low exposure: adults spending less than 50% of time in surface waters with no planktonic stage.
- Moderate exposure: adults spending less than 50% of time in surface waters with planktonic stage.
- High exposure: adults spending more than 50% of time in surface waters with planktonic stage.

Examples: *Centroscymnus coelolepis* is a deep-water species with no planktonic stage, so it should be classified as low exposure. *Sarda sarda* is a deep-water species with planktonic stage, so it should be classified as moderate exposure. *Dicentrarchus labrax* occurs in coastal habitats and has planktonic stage, so it should be classified as high exposure.

5. Extreme events.

Extreme events frequency projections have high uncertainty in scenarios of climate change (Hoegh-Guldberg et al., 2018). Nevertheless, the frequency of these events has already been observed to increase in Portugal with seasonal variations (Santo et al., 2014). This could impact the ocean ecosystem by means of modifications on the river runoff patterns, or affecting the depth of the mixing surface layers by increased storminess, with consequences on the nutrient recycling or the establishment of clines, pivotal for planktonic organisms. Highly mobile organisms, deep water organisms, or organisms with direct development are expected to be less sensitive to changes in the frequency of these events, as they will be able to avoid these events.

- Low exposure: adults spending less than 50% of time in surface waters with no planktonic stage.
- Moderate exposure: adults spending less than 50% of time in surface waters with planktonic stage.
- High exposure: adults spending more than 50% of time in surface waters with planktonic stage.

<u>Examples:</u> *Centroscymnus coelolepis* is a deep water species with no planktonic stage, so should be classified as low exposure. *Lophius budegassa* is a deep water species with planktonic larvae, so should be classified as moderate exposure. *Diplodus vulgaris* is a shallow water species with planktonic larvae, so should be classified as high exposure.

6. Riverflow / rainfall.

Riverflow is expected to decrease both under scenarios RCP4.5 and RCP8.5 in Portugal, especially in the south region. Riverflow is responsible for nutrient runoff in coastal waters promoting primary production in spawning areas and has been found to be related to marine fish landings (Bueno-Pardo et al., 2020). Species depending on estuarine or freshwater habitats for reproduction or spawning are more vulnerable than open ocean species.

- Low exposure: no life phase associated to estuarine or freshwater habitats.
- Moderate exposure: some life phase (larva, juvenile, adult) associated to estuarine or freshwater habitats.
- High exposure: life cycle enterely or almost enterely dependent on estuarine or freshwater habitats.

Examples: *Thunnus alalunga* life cycle is totally independent on estuarine or freshwater habitats, so it should be classified as low exposure. *Diplodus sargus* juveniles are dependent on estuarine and coastal lagoons, but not the adults, so it should be classified as moderate exposure. *Chelon auratus* life cycle is almost entirely dependent on estuarine habitats, so should be classified as high exposure.

7. Plankton productivity.

Both primary production and zooplankton biomass production are expected to increase in Portuguese waters between 2040 and 2050, especially under scenario RCP8.5. This increase would be beneficial for organisms with planktonic larvae if the timing of planktonic blooms is not mismatched and the increase of currents does not push the planktonic productivity offshore. Filter feeding species and species with planktonic larvae are highly exposed to these variations.

- Low exposure: species with direct development or larvae entirely dependent on yolk reserves.
- Moderate exposure: species with planktonic larvae that use both yolk reserves and planktonic food.
- High exposure: species with planktonic larvae dependent on phyto or zooplankton for development, or filter-feeding adults.

Examples: *Leucoraja naevus* does not have a planktonic larval stage, so should be classified as low exposure. *Salmo salar* larvae have big yolk sacs, so they are somehow independent on planktonic items as food, it should be classified as moderate exposure. *Donax trunculus* is a filter-feeding species, so should be classified as high exposure.

Attributes related to the species’ sensitivity to climate change.

Sensitivity indicators represent biological traits related to the ability of a species to respond to environmental change (Hare et al., 2016).

8. Trophic level.

Higher trophic level organisms have lower individual and population growth rates than producers and primary or secondary consummers. Also, higher trophic level organisms synchronize the timing of reproduction to match the periods of high abundance of primary producers in temperate and high latitudes (Durant et al., 2019). In consequence, environmental change, or variations on the timing of peak abundances of primary producers can exert a big influence on higher trophic levels with longer generation times (slower population response), or if the timing of reproduction is mismatched between groups. For these reasons, high trophic level species are more sensitive to environmental change than low trophic level species.

- Low exposure: low trophic levels (between 2 and 3).
- Moderate exposure: intermediate trophic levels (between 3 and 4).
- High exposure: high trophic levels (higher than 4).

<u>Examples:</u> *Necora puber* has a trophic level of 2.72, so it should be classified as low sensitivity. *Homarus gammarus* has a trophic level of 3.7, so it should be classified as moderate sensitivity. *Beryx decadactylus* has a trophic level of 4.13 so ti should be classified as high sensitivity.

9. Fecundity.

Species with higher fecundity should be less sensitive to climate change than species with low fecundity due to their enhanced reproductive output, connectivity between populations, and ability to reach and colonize different areas. In a similar approach to assess climate change sensitivity of Australian species, Pecl et al. (2014) classified fecundity in three groups: >20,000, 100-200,000, and <100 eggs per year, representing low, moderate and high sensitivity respectively. Here we adjusted the fecundity categories in regards of the biology of Portuguese species.

- Low sensitivity: more than 20,000 eggs per female per year.
- Moderate sensitivity: between 1,000 and 20,000 eggs per female per year.
- High sensitivity: less than 1,000 eggs per female per year.

<u>Examples:</u> *Sardina pilchardus* spawns around 80,000 eggs, so it should be classified as low sensitivity. *Belone belone* spawns between 1,000 and 35,000 eggs, so it should be classified as moderate sensitivity. *Galeus melastomus* lays less than 100 eggs, so it should be classified as high sensitivity.

10. Semelparity/Iteropary.

Semelparous species die short after reproductive events, while iteroparous species pass through several reproductive events during their lifespan. Due to their lower reproductive output, semelparous species are more sensitive to climate change than iteroparous species.

- Low sensitivity: iteroparous species.
- Moderate sensitivity: few reproductive events (<5) during its lifespan.
- High sensitivity: semelparous.

Examples: *Sardina pilchardus* is able to reproduce several times during its entire lifespan, so it should be classified as low sensitivity. *Sepia officinalis* reproduces few times before dying, so it should be classified as moderate sensitivity. *Octopus vulgaris* dies after a single reproduction event, so it should be classified as high sensitivity.

11. Egg spawning strategy.

Parental care enhances the survival of the brood in the marine environment. Although it is usually related to lower fecundities (Charnov and Ernest, 2006), individual youngs under parental care are less sensitive to environmental change.

- Low sensitivity: egg bearers, or species with internal fecundity and development.
- Moderate sensitivity: eggs attached to substrate.
- High sensitivity: broadcast spawners.

Examples: *Prionace glauca* has internal fecundity and gives birth youngs, so it should be classified as low sensitivity. *Raja clavata* has internal fecundity, but their eggs are attached to a substrate where they finish development, so it should be classified as moderate sensitivity. *Sardina pilchardus* broadcasts its eggs in the water column, so it should be classified as high sensitivity.

12. Von Bertalanffy K.

The von Bertalanffy growth curve describes the length (L) at age *a* of an organism as:

$L(a) = L_{inf}\,(1-e^{-K(a-a0)})$

where L_{inf} is the asymptotic size, a_0 is a parameter related to size at age 0 and *K* is the growth coefficient. *K* represents how fast individuals grow (the higher *K* the faster they grow, as it is expressed in reciprocal time units, $year^{-1}$). It is related to sensitivity to environmental change in a manner that slow growers (low *K*) should be more sensitive to climate change than fast growers (high *K*) because of their longer generation times, higher age at maturity and lower fecundities.

- Low sensitivity: K > 0.30.
- Moderate sensitivity: K between 0.20 and 0.30.

- High sensitivity: K < 0.20.

Examples: *Pegusa lascaris* has *K*=0.39 so it should be classified as low sensitivity. *Boops boops* has *K*=0.24 so it should be classified as moderate sensitivity. *Trachurus picturatus* has *K*=0.18 so should be classified as high sensitivity.

13. Age at maturity.

Age at maturity relates to generation time of the species, and consequently to its growth rate. Species with slow maturation are more sensitive to climate change than species with fast development due to their lowered reproductive output.

- Low sensitivity: <2 years.
- Moderate sensitivity: 2-10 years.
- High sensitivity: >10 years.

Examples: *Pagellus erythrinus* matures at age 1, so it should be classified as low sensitivity. *Lithognathus mormyrus* matures at age 2, so it should be classified as moderate sensitivity. *Helicolenus dactylopterus* matures at age 15, so it should be classified as high sensitivity.

14. Population growth rate (r).

The population growth rate is related to different life histories such as individual growth, maturation, fecundity and mortality rates. Hence, species with high population growth rates are less sensitive to climate change.

- Low sensitivity: r > 0.50.
- Moderate sensitivity: r between 0.50 and 0.20.
- High sensitivity: r < 0.20.

Examples: *Dicentrarchus labrax* has r=0.54, so it should be classified as low sensitivity. *Conger conger* has r=0.35, so it should be classified as moderate sensitivity. *Raja clavata* has r=0.18, so it should be classified as high sensitivity.

15. Longevity.

This is related to growth and developmental patterns, with higher longevity related to slow growth and development. Organisms with longer longevity are more sensitive to environmental change because of their larger generation time and lower overall reproductive output.

- Low sensitivity: 1-4 years.
- Moderate sensitivity: 5-9 years.
- High sensitivity: >10 years.

Examples: *Sepia officinalis* lifespan is around 2 years, so it should be classified as low sensitivity. *Scorpaena notata* lifespan is around 4 years, so it should be classified as moderate sensitivity. *Helicolenus dactylopterus* lifespan reach 43 years, so it should be classified as high sensitivity.

16. Gonochorism.

Gonochorism refers the fact of species having separated sexes. The reproductive output of these species is highly dependent on the encounter rate between sexual compatible individuals. In this context, hermaphrodite species are expected to have higher encounter rates between sexual compatible individuals, increasing the probabilities of having successful reproductive encounters with other individuals in comparison to gonochorist species, lowering their sensitivity to environmental change. Another existing option are protandry (life starts as male and ends as female), or protogyny (life starts as female and ends as male) which are intermediate strategies that could be considered as indicators of moderate sensitivity as their encounter rates are expected to be intermediate between gonochorist and hermaphrodite species.

- Low sensitivity: Hermaphrodite.
- Moderate sensitivity: Protogyny or Protandry.
- High sensitivity: Gonochorist or dioecian species.

Examples: *Homarus gammarus* is an hermaphrodite species, so it should be clasified as low sensitivity. *Spondyliosoma cantharus* strategy is protogyny, so it should be classified as moderate sensitivity. *Sepia officinalis* is gonochorist (dioecism) so it should be classified as high sensitivity.

17. Spawning cycle.

Larvae or juveniles of species spawning during a narrow period of time are more likely to be affected by a mismatch with peak abundances of resources eventually favouring the survival of youngs. In the context of environmental change, species with a narrow spawning cycle should be classified as high sensitive, while species with a wide spawning cycle should be classified as low sensitive.

- Low sensitivity: spawning occurs throughtout the year.
- Moderate sensitivity: there are two or more spawning peaks per year.
- High sensitivity: there is only one spawning peak per year or the species only reproduces once in a lifetime.

Examples: *Thunnus albacares* spawns throughout the year, so it should be classified as low sensitivity. *Pagellus erythrinus* has two spawning peaks per year, so it should be classified as moderate sensitivity. *Spondyliosoma cantharus* has only one spawning peak per year, so it should be classified as high sensitivity.

18. Planktonic larval duration (PLD).

The planktonic larval phase is the period of higher dispersive potential for most marine species. Longer larval duration is related to higher connectivity between populations as larvae wander for longer periods of time, having also higher potential to colonize new areas. Although the planktonic stage is also the period of life

with the highest instantaneus mortality rate of marine organisms, Jablonski & Lutz (1983) found that marine invertebrates with relatively long planktonic stage are more persistent in the fossil record than species with no planktonic stage. In this way, species with longer PLD are less sensitive to climate-driven environmental changes.

- Low sensitivity: PLD longer than 21 days.
- Moderate sensitivity: PLD between 7 and 21 days; or species with no planktonic larvae.
- High sensitivity: PLD shorter than 7 days.

Examples. *Scophthalmus rhombus* has a PLD of 26 days, so it should be classified as low sensitivity. *Micromesistius poutassou* has a PLD of around 20 days, so it should be classified as moderate sensitivity. *Mustelus asterias* has no planktonic larval stage, so it should be classified as high sensitivity.

19. Latitudinal range.

The latitudinal range of a species relates to the range of environmental variables tolerated, being that species with narrow latitudinal ranges are more dependent on specific environmental conditions. In the event of environmental change, species with a closer dependence of local conditions are more vulnerable to the changes. Similarly,

- Low sensitivity: latitudinal range > 90º
- Moderate sensitivity: latitudinal range between 45 and 90º
- High sensitivity: latitudinal range < 45º

Examples: *Octopus vulgaris* has a latitudinal range of occurrence of 95º, so it should be classified as low sensitivity. *Trisopterus luscus* has a latitudinal range of occurrence of 37º, so it should be classified as moderate sensitivity. *Chelon auratus* has a latitudinal range of occurrence of 44º, so it should be classified as high sensitivity.

20. Temperature range.

Stenotherm organisms (organisms with a narrow range of temperature tolerance) are more sensible to changes in temperature because their plasticity to adapt to changes in temperature is expected to be more restricted than that of euritherm organisms (organisms with a wide range of temperature tolerance).

- Low sensitivity: temperature range > 20 ºC
- Moderate sensitivity: temperature range between 10 and 20 ºC
- High sensitivity: temperature range < 10 ºC

Examples: *Necora puber* has a preferred temperature range of 24ºC, so it should be classified as low sensitivity. *Sepia officinalis* has a preferred temperature range of 11.3ºC, so it should be classified as moderate sensitivity. *Lophius budegassa* has a preferred temperature range of 4.5 ºC, so it should be classified as high sensitivity.

21. Adult mobility.

The mobility of a given species during the adult phase is related to its ability to avoid extreme events and environmental changes. Less mobile species are more sensitive to climate change than species with higher mobility.

- Low sensitivity: pelagic species.
- Moderate sensitivity: demersal species.
- High sensitivity: sessile or benthic species.

Examples: *Sardina pilchardus* is a pelagic species, so it should be classified as low sensitivity. *Scyliorhinus canicula* is a demersal species, so it should be classified as moderate sensitivity. *Spisula solida* is a benthic species, so it should be classified as high sensitivity.

22. Seasonal migrations or reproductive movements.

Species performing seasonal or spawning migrations are more vulnerable to climate change because of their dependence on different habitats to complete their life cycle, having that perturbations in one of the habitats used by a species could have negative consequences (Pecl et al., 2014). In this manner, species performing migrations are more sensitive to climate change than non-migrant species.

- Low sensitivity: species not performing any seasonal or reproductive migration.
- Moderate sensitivity: species performing reproductive movements between two different habitats, implying displacements of no more than 3º of latitude or longitude.
- High sensitivity: species performing seasonal migrations comprising displacements of more than 3º of latitude or longitude.

Examples: *Spisula solida* is a benthic species not performing any seasonal or reproductive movement, so it should be classified as low sensitivity. *Pagellus bogaraveo* adults move towards the coast from the continental slope to spawn, so it should be classified as moderate sensitivity. *Aphanopus carbo* life cycle involves a large scale migration, so it should be classified as high sensitivity.

23. Species sociability.

Some species live in groups, which provides them with more opportunities to detect the presence of predators or changes in the environment. Species living in groups also have higher encounter rates between individuals of different sex, favouring their reproduction. In this way, species forming schools are less sensitive to climate change than solitary species.

- Low sensitivity: forms shoals (> 1000 individuals aprox.).
- Moderate sensitivity: gregarious species (>10 individuals aprox.).
- High sensitivity: lives in small groups (<10 individuals) or solitary.

Examples: *Sardina pilchardus* forms shoals, so it should be classified as low sensitivity. *Boops boops* is a gregarious species but it does not forms big shoals, so it should be classified as moderate sensitivity. *Cerastoderma edule* lives in habitats where many individuals are found, however, they do not live as a group since its capacity of interaction is very low, so it should be classified as high sensitivity.

24. Complexity of reproductive strategy.

Species with complex reproductive strategies, courtship displays, reproductive behaviour, internal fecundity, parental guarding strategy, etc. are more likely to be affected by environmental change through interferences with their reproduction. Most of these traits require specific environmental cues potentially affected by in the context of environmental change.

- Low sensitivity: organisms with external fecundity without any special reproductive behaviour, courtship, or specific environmental conditions for reproduction or settlement.
- Moderate sensitivity: organisms with external fecundity with some special reproductive behaviour.
- High sensitivity: organisms with external fecundity depending on some specific environmental cue for reproduction, or species with internal fecundity.

Examples: *Sardina pilchardus* is a species with external fecundity with no special requirements for reproduction, so it should be classified as low sensitivity. *Sepia officinalis* is a species with external fecundity but with a complex courtship display and egg guarding strategy, so it should be classified as moderate sensitivity. *Prionace glauca* is a species with internal fecundity, so it should be classified as high sensitivity.

Attributes related to the species' adaptive capacity to climate change.

The adaptive capacity of a species refers to its resilience to modifications of the environment (Glick et al., 2011). Here we propose four indicators considering the vulnerability of the species or the stocks together with general aspects of the ecology of the species.

25. ICES stock status.

The International Council for the Exploitation of the Sea (ICES) categorizes the status of exploited stocks in relation to reference points. These categories can be informative on how healthy a given stock is, providing valuable information on the species adaptive capacity. The categories considered are: 1) stock sustainably fished: desirable situation, e.g. fishing pressure is below the relevant reference point or stock size is above the relevant reference point; 2) undefined: status lies between the precautionary and limit reference points; 3) overfished or on the verge of overfishing: undesirable situation, e.g. fishing pressure is above the relevant reference point or stock size is below the relevant reference point; 4) unknown: the status of the stock is either unknown when neither quantitative assessment nor proxy calculation exist, or undefined when there is an analytical assessment but reference points are undefined. The adaptive capacity of overfished species is lower because larger individuals, or even juveniles, could be removed from the population.

- Low adaptive capacity: overfished stock.

- Moderate adaptive capacity: undefined or unknown stock status.

- High adaptive capacity: stock sustainably fished.

<u>Examples:</u> *Merluccius merluccius* stock is overexploited, so it should be considered as low adaptive capacity. *Zeus faber* stock status is unknown, so it should be considered as moderate adaptive capacity. *Lophius budegassa* is considered a sustainably exploited stock, so the adaptive capacity should be considered as high.

26. Replenishment potential.

R-strategist species are fast growing organisms with high fecundities and short lifespan, while k-strategist are slow growing organisms producing few offspring and long lifespan. In this manner, r-strategist have higher adaptive capacity to environmental change than k-strategists because of their enhanced reproductive output, population growth rate and faster generation times.

- Low adaptive capacity: k-strategist: late maturing, slow growth and few young.
- Moderate adaptive capacity: intermediate strategy.
- High adaptive capacity: r-strategist: early maturing, fast growth and many youngs.

Examples: *Prionace glauca* is a species maturing late, slowly growing and with low fecundity, so it should be classified as low adaptive capacity. *Merluccius merluccius* is a species with intermediate characteristics, so it should be classified as moderate adaptive capacity. *Sardina pilchardus* is a species with short lifespan, fast growth and high fecundity, so it should be classified as high adaptive capacity.

27. Vulnerability according to the International Union for Conservation of Nature (IUCN).

Vulnerability is defined by the IUCN as "A set of conditions and processes resulting from physical, social, economic and environmental factors, indicating the susceptibility of a community to the impact of hazards (ISDR 2004)". A taxon is classified as vulnerable when facing high risk of extinction in the wild in the immediate future. Hence, vulnerable species have a lowered adaptive capacity to environmental change.

- Low adaptive capacity: species classified as extinct, extinct in the wild, critically endangered, endangered, or vulnerable.
- Moderate adaptive capacity: species classified as near threatened or as data deficient.
- High adaptive capacity: species classified as least concern.

Examples: *Umbrina cirrosa* is classified as vulnerable, so it should be considered as a species with low adaptive capacity. *Raja undulata* is classified as near threatened, so it should be considered as a species with moderate adaptive capacity. *Solea solea* is classified as least concern, so it should be considered as a species with high adaptive capacity.

28. Vulnerability to fisheries.

Cheung et al. (2005) provided a numeric estimation ranging from 0 to 100 of the species' vulnerability to fisheries. Exploited species that are more vulnerable to fisheries are subject to a high stress and hence have a lowered adaptive capacity to face environmental changes.

- Low adaptive capacity: high vulnerable species (values higher or equal than 66).
- Moderate adaptive capacity: moderately vulnerable species (numeric values between 33 and 66).
- High adaptive capacity: low vulnerable species (values lower or equal than 33).

Examples: *Conger conger* has a vulnerability of 85 so it should be classified as low adaptive capacity. *Thunnus obesus* has a vulnerability to fisheries of 55, so it should be classified as moderate adaptive capacity. *Maja squinado* has a vulnerability to fisheries of 12, so it should be classified as high adaptive capacity.

29. Fishing pressure

The ecological vulnerability of commercial species is dependent, to a variable extent, on the pressure exerted by fisheries. These activities can remove a considerable proportion of individuals of specific ages shaping the structure of the population and its main dynamics. Exploited species are, in consequence, less resilient to environmental change.

The mean size of the catch is ussually used as an indicator of the pressure exerted by fisheries on a given population. Commercial fisheries in early stages of exploitation catch large individuals with higher economic value. However, as the exploitation of the stock continues and large individuals are removed, a trend to capture smaller individuals is often observed. The size of the catch by Portuguese fisheries is reported by official data from the DGRM in a 1-6 code, where 1 represents large mature individuals of appropriate size and 6 represents individuals below the recommended minimum catch size (see columns on Portuguese fisheries in the Summary Sheet).

In addition to this information, other indicators such as the selectivity of the gears deployed (non selective gears capture young individuals and/or destroy the species' habitat), or specificity of the fisheries capturing a given species (species with associated fishery are more vulnerable than by-catch species) can be considered.

- Low adaptive capacity: mean size of the catch > 3, or species captured by non-selective gears (i.e. trawling), or species targeted by an specific fishery.
- Intermediate adaptive capacity: mean size of the catch between 2 and 3, or species captured by semi-selective gears (i.e. purse-seine), or species targeted by a fishery shared with other few species.
- High adaptive capacity: mean size of the catch < 2, or species captured by selective gears (i.e. traditional or multi-gear), or by-catch species.

Examples: *Merluccius merluccius* mean size of the catch is 3.80 in the North, while it is captured by the multi-gear and trawling *métiers*, so it should be classified as low adaptive capacity. *Lepidopus caudatus* mean size of the catch is 2.30 in the North, while it is mainly captured by muti-gear *métiers*, so it should be classified as intermediate adaptive capacity, with some tallies also allocated to high adaptive capacity. *Brama brama* mean size of the catch is 1.71 in the North, while it is captured by multi-gear *métiers*, so it should be classified as high adaptive capacity.

Directional effects of climate change.

The panel of experts was asked to evaluate the information available in the literature relating the species interdependence on environmental variables integrating the knowledge from experiments, models, observations, etc. The objective was to provide a formed decision on the most likely directional (positive, negative or neutral) effect of climate change on the distribution and productivity of the species under consideration. The expected evolution of environmental variables under the scenarios of climate change is pivotal for making these assessments, so the outputs from the physical-biogeochemical model were shown to experts in order to support their decission.

30. Predicted directional effect of climate change.

Based on results from experiments, models, or observations, the experts should classify the species considering the impact of the expected future environmental condition and the knowledge on the biology and ecology of the species.

- Negative: the species productivity or distribution in Portuguese waters will probably be negatively affected by future environmental conditions.
- Neutral: neither the species productivity nor distribution will be negatively or positively affected by future environmental conditions, or there is no data to make any strong prediction for the future.

Positive: the species productivity or distribution in Portuguese waters will probably be positively affected by future environmental conditions.

Authors were encouraged to perform a bibliographic search using the Web of Knowledge introducing the latin name of the species under consideration followed by keywords related to environmental variables such as “temperature”, “pH”, “salinity”, or to ecological processes such as “recruitment”, “feeding”, “reproduction”. A general search including the keywords “climate change”, “environmental variables” and “Portugal” was also recommended. Ideally this search was restricted to the period 2000-2020.

In the case of different variables having contradictory effects on the biology or ecology of the species, the experts should decide how to distribute the tallies among the three bins accordingly.

Examples:

- Negative effects: *Octopus vulgaris*. Positive effect of SST on pre-recruit distribution and landings has been described (Gamito et al., 2015). However, in the south coast of Portugal, the effect is reversed and SST could be negatively related to landings of octopus in this region (Gamito et al., 2016). The same in Tunisia (Chédia et al., 2010). Weight losses and mortality were recorded when temperature increased 3ºC: their temperature range seems to be a bit narrow for some areas in the Mediterranean (Aguado and Barcia, 2003). Temperature increase of 3ºC caused an increase in embryonic metabolic consumption (Lopex et al., 2016). Experiments in Portugal show 30% mortality increase with +3ºC. The percentage of premature paralarvae also will increase, affecting connectivity and dispersal ability. Premature paralavae increased, from 0% to 18% by increasing temperature from 18 to 21ºC (Repolho et al., 2014).

- Neutral effects: *Sepia officinalis*. Both temperature and pH have distinct effects on the bioaccumulation of radionuclides and metals in cuttlefish embryos, but these effects seem to be compensated by animal physiology (Lacoue-Labarthe et al., 2012). Landings have been positively related to SST in the southwest coast of Portugal (Gamito et al., 2015). Low genetic interpopulation variability (Shaw and Pérez-Losada, 2000). Adult sepia seems to be resilient to long term increased pCO_2 (Gutowska et al., 2008). Embryos more resilient to ocean warming than *Loligo sp.* (Pimentel et al., 2012).

- Positive effects: *Engraulis encrasicolus.* Temperature and zooplankton biomass are expected to enhance growth and fecundity of this short-living species (Taboada and Anadón, 2016). Fisheries are an important determinant of the species biomass together with environmental variables. The implementation of harvest control rules seems to be adequate in anchovy population control and could have avoided the population collapse of 2005 in the Bay of Biscay (Bueno-Pardo et al., 2019).

Data quality.

Data quality is evaluated by the experts in a four-level ranking (0-3) according to these criteria:

0: No Data. No information to base an attribute score on. Very little is known about the species or related species and there is no basis for forming an expert opinion.

1: Expert Judgement. The attribute score reflects the expert judgement of the reviewer and is based on their general knowledge of the species, or related species, and their relative role on the ecosystem.

2: Limited Data. The score is based on data which has a higher degree of uncertainty. The data used to score the attribute may be based on related or similar species, come from outside the study area or the reliability of the source may be limited.

3: Adequate Data. The score is based on data which have been observed, modelled or empirically measured for the species in question and comes from a reputable source.

Bibliography.

- Bakun, A.; Black, B. A.; Bograd, S. J.; García-Reyes, M.; Miller, A. J.; Rykaczewski, R. R.; Sydeman, W. J. (2015) Anticipated effects of climate change on coastal upwelling ecosystems. Current Climate Change Reports 1: 85-93.
- Bueno, J; López-Urrutia, A. (2012) The offspring-development-time/offspring-number trade-off. The American Naturalist 179, No 6.
- Bueno-Pardo, J.; Petitgas, P.; Kay, S.; Huret, M. (2019) Integration of bioenergetics in an individual-based model to hindcast anchovy dynamics in the Bay of Biscay. ICES Journal of Marine Science, doi:10.1093/icesjms/fsz239.
- Bueno-Pardo, J.; Pierce, G. J.; Cabecinha, E.; Grilo, C.; Assis, J.; Valavanis, V.; Pita, C.; Dubert, J.; Leitão, F.; Queiroga, H. (2020) Trends and drivers of marine fish landings in Portugal since its entrance in the European Union. ICES Journal of Marine Science, doi 10.1093/icesjms/fsaa010.
- Charnov, E. L.; Ernest, S. K. M. (2006) The offspring-size/clutch-size trade-off in mammals. The American Naturalist 167: 578-582.
- Cheung, W. W. L.; Pitcher, T. J.; Pauly, D. (2005) A fuzzy logic expert system to estimate intrinstic extinction vulnerabilities of marine fishes to fishing. Biological Conservation 124: 97-111.
- Cushing, D. H. (1990) Plankton production and year-class strength in fish populations: an update of the match-mismatch hypothesis. Advances in Marine Biology 26: 249-293.
- Dubert
- Durant, J. M.; Molinero, J. C.; Ottersen, G.; Reygondeau, G.; Stige, L. C.; Langangen, Ø. (2019) Contrasting effects of rising temperatures on trophic interactions in marine ecosystems. Scientific Reports 9: 15213.
- Glick, P.; Stein, B. A.; Edelson, N. A. (2011) Scanning the Conservation Horizon: a guide to climate change vulnerability assessment, Draft. National Wildlife. National Widllife Federation, Washington, DC.
- Gillooly, J. F., Charnov, E. L.; West, G. B.; Savage, V. M.; and Brown, J. H. (2002) Effects of size and temperature on developmental time. Nature 417:70–73.
- Hoegh-Guldberg, O.; Jacob, D; Taylor, M.; et al. (2018) Impacts of 1.5ºC global warming on natural and human systems. In: Global warming of 1.5ºC. An IPCC special report on the impacts of global warming of 1.5ºC above pre-industrial levels and related global greenhouse gas emission pathways, in the context of strengthening the global response to the threat of climate change, sustainable development, and efforts to eradicate poverty [Masson-Delmotte, V., P. Zhai, H.-O. Pörtner, D. Roberts, J. Skea, P.R. Shukla, A. Pirani, W. Moufouma-Okia, C. Péan, R. Pidcock, S. Connors, J.B.R. Matthews, Y. Chen, X. Zhou, M.I. Gomis, E. Lonnoy, T. Maycock, M. Tignor, and T. Waterfield (eds.)].
- Kinne, O. (1966) Physiological aspects of animal life in estuaries with special reference to salinity. Netherlands Journal of Sea Research 3: 222-244.
- Leitão, F.; Baptista, V.; Vieira, V.; Silva, P. L.; Relvas, P.; Teodósio, M. A. (2019) A 60-year time series analyses of the Upwelling along the Portuguese coast. Water 11: 1285.
- Pecl, G. T.; Ward, T. M.; Doubleday, Z. A.; Clarke, S.; Day, J.; Dixon, C.; Frusher, S.; Gibbs, P.; Hobday, A. J.; Hutchinson, N.; Jennings, S.; Jones, K.; Li, X.; Spooner, D.; Stoklosa, R. (2014). Rapid assessment of fisheries species sensitivity to climate change. Climatic Change, doi 10.1007/s10584-014-1284-z.

- Santo, F. E.; Ramos, A. M.; Lima, M. I.; Trigo, R. M. (2014) Seasonal changes in daily precipitation extremes in mainland Portugal from 1941 to 2007. Regional Environmental Change 14: 1765-1788.
- Taboada, F. G.; Anadón, R. (2016) Determining the causes behind the collapse of a small pelagic fishery using Bayesian population modelling. Ecological Applications, 26: 886-898.
