# Supplementary material for: Climate change vulnerability assessment of the main marine commercial fish and invertebrates of Portugal
Source: Sci Rep. 2021 Feb 3;11:2958. doi: 10.1038/s41598-021-82595-5 (PMC7858592; doi:10.1038/s41598-021-82595-5)
Supplement: Supplementary file 3 — Supplementary Information 3. [file 41598_2021_82595_MOESM3_ESM.pdf]

Climate change vulnerability assessment of the main marine commercial fish and invertebrates of Portugal

SUPPLEMENTARY INFORMATION 3:

Expected environmental change in the context of climate change

Juan Bueno-Pardo[1*], Daniela Nobre[1], João N. Monteiro[1], Pedro M. Sousa[1], Eudriano F. S. Costa[1], Vânia Baptista[1], Andreia Ovelheiro[1], Vasco M. N. C. S. Vieira[2], Luís Chícharo[3], Miguel Gaspar[4], Karim Erzini[1], Susan Kay[5], Henrique Queiroga[6], Maria A. Teodósio[1], Francisco Leitão[1]

[1] Centro de Ciências do Mar (CCMAR), Universidade do Algarve, Campus de Gambelas, Faro 8005-139, Portugal

[2] Instituto Superior Técnico, Lisboa 1041-001, Portugal

[3] Faculdade de Ciência e Tecnologia, Universidade do Algarve, Campus de Gambelas, Faro 8005-139, Portugal

[4] Instituto Português do Mar e a Atmósfera (IPMA), Centro de Olhão, Olhão 8700-305, Portugal

[5] Plymouth Marine Laboratory, Prospect Place, The Hoe, Plymouth PL1 3DH, UK

[6] Departamento de Biologia e Centro de Estudos do Ambiente e do Mar (CESAM), Universidade de Aveiro, Campus Universitário de Santiago, Aveiro 3810-193, Portugal

*** Corresponding author: jbuenopardo@gmail.com**

The outputs of the physical-biogeochemical model POLCOMS-ERSEM (see methods in the main text) were used to estimate the degree of change of the main physical variables with influence on the vital cycles of marine organisms in upwelling-driven systems (Bakun et al., 2015). Here we show the anomalies in the environmental conditions in Portuguese waters between the future period (2040-2059) and the reference period (2000-2019). The values shown represent the difference between period averages, calculated based on yearly averages. Two scenarios of climate change are considered for the future: RCP 4.5 and RCP 8.5. Table 2 of the main text compiles these data and shows the averages for each region, time slice and climate change scenario, with the respective percentage of variation calculated.

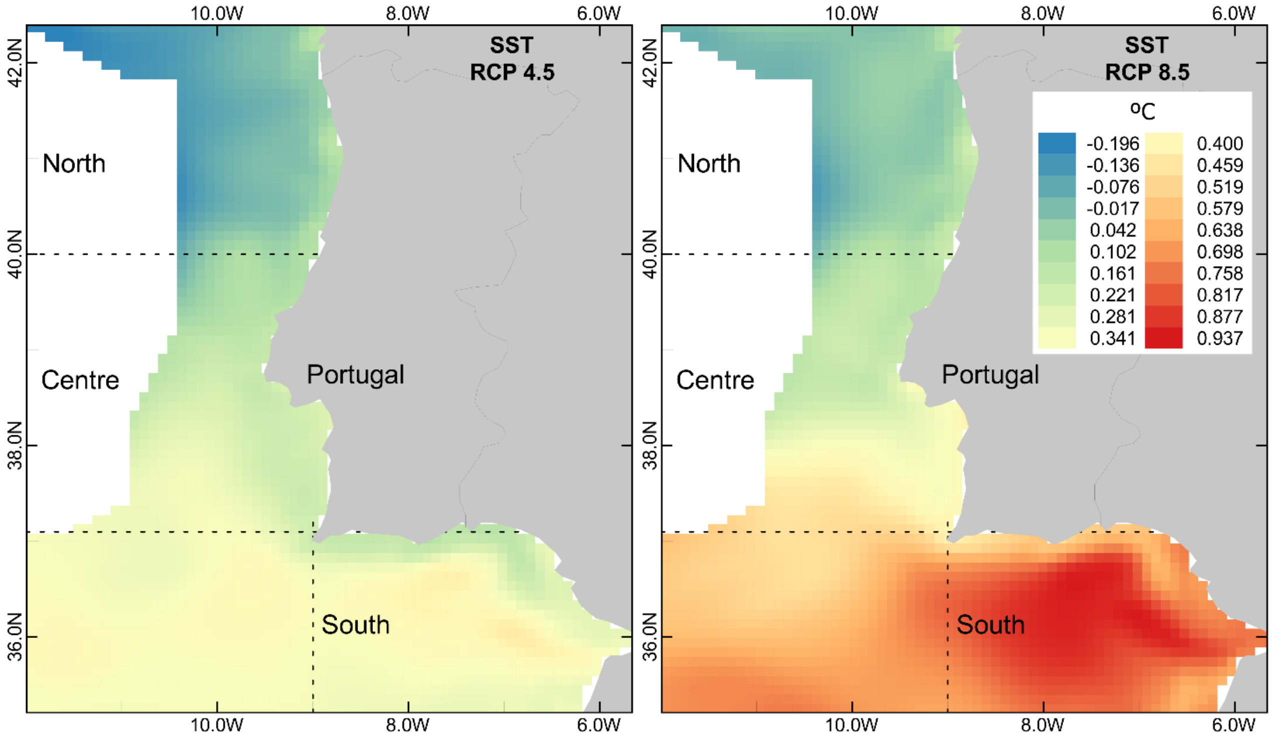

Figure SI3-1. Sea surface temperature (SST) anomaly calculated based on period averages (2040-2059 vs 2000-2019) for scenario RCP 4.5 (left), and scenario RCP 8.5 (right).

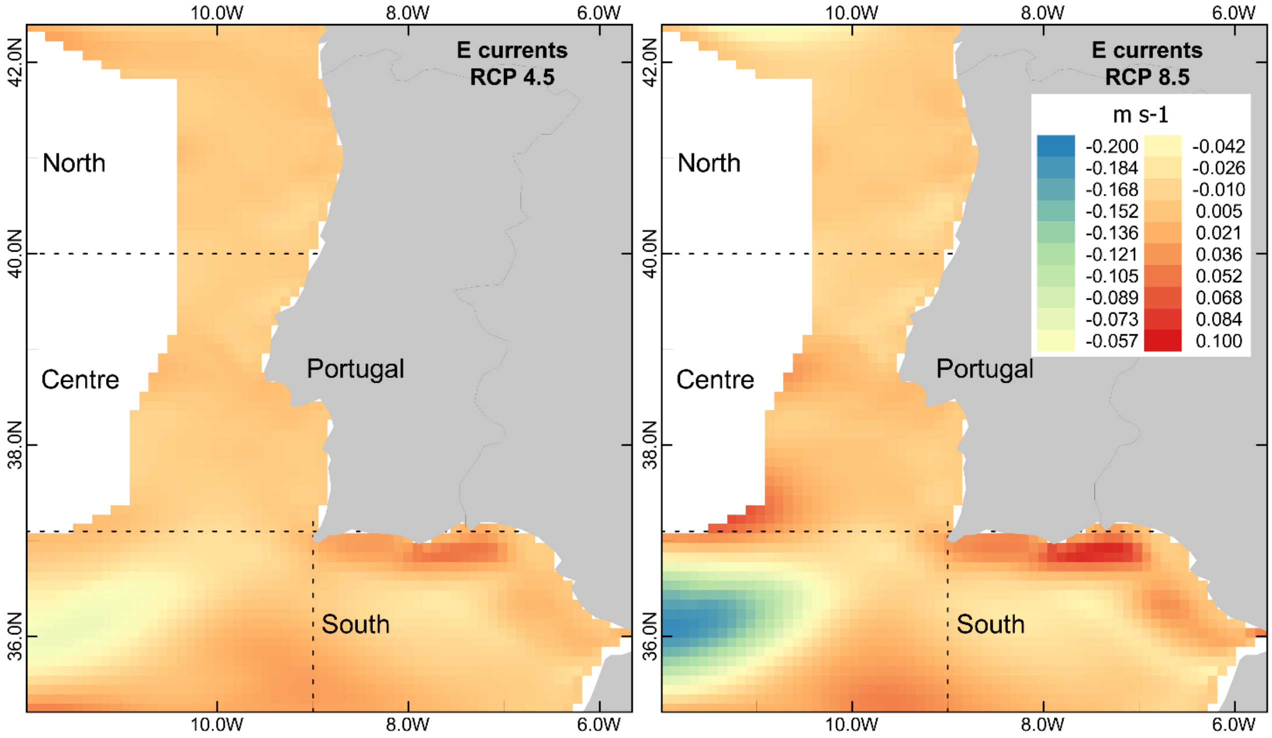

Figure SI3-2. Eastward current velocity anomaly (m s^{-1}) in surface, calculated based on period averages (2040-2059 vs 2000-2019) for scenario RCP 4.5 (left), and scenario RCP 8.5 (right).

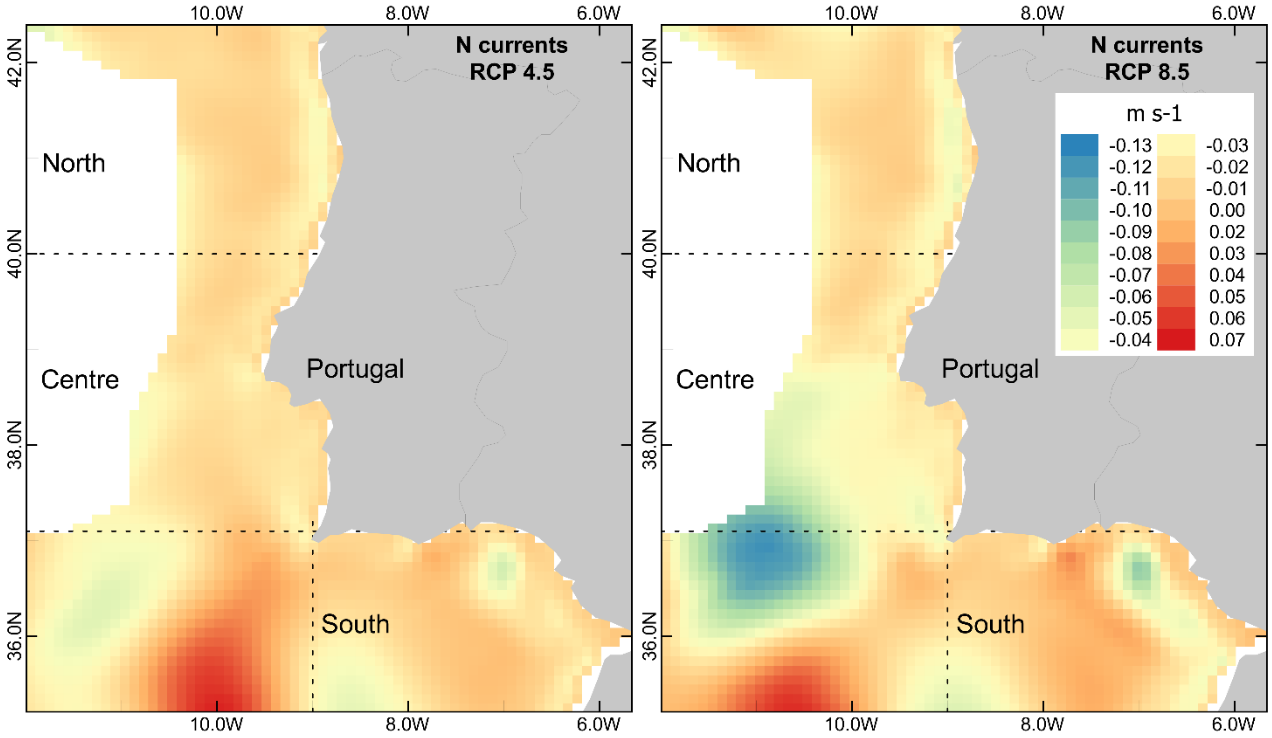

Figure SI3-3. Northward current anomaly (m s^{-1}) in surface, calculated based on period averages (2040-2059 vs 2000-2019) for scenario RCP 4.5 (left), and scenario RCP 8.5 (right).

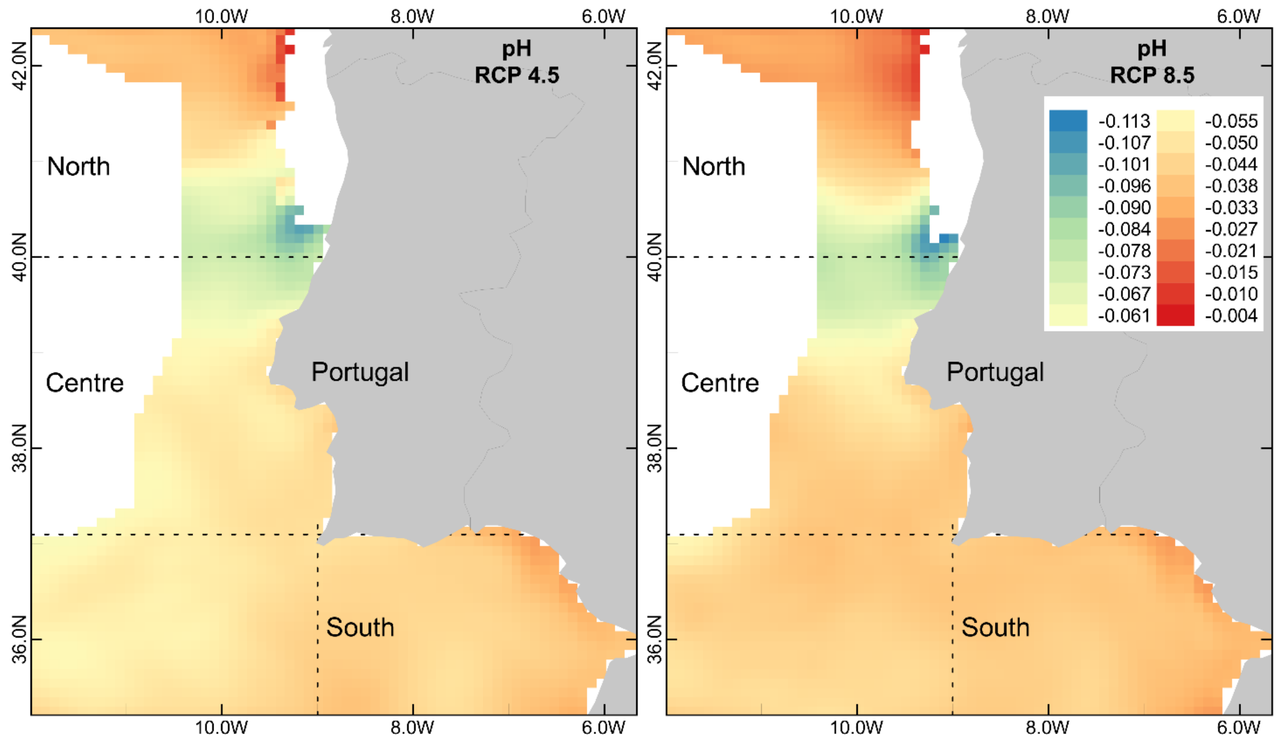

Figure SI3-4. Surface pH anomaly calculated based on period averages (2040-2059 vs 2000-2019) for scenario RCP 4.5 (left), and scenario RCP 8.5 (right).

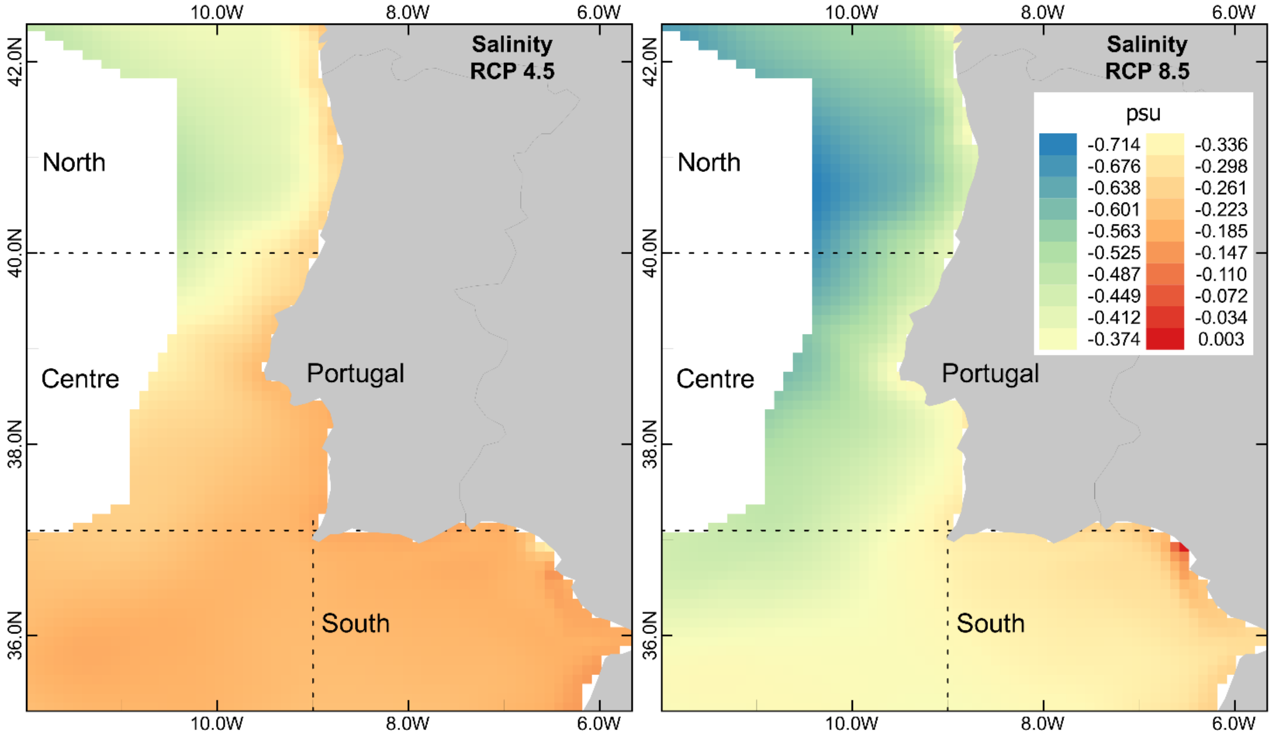

Figure SI3-5. Surface salinity anomaly (psu) calculated based on period averages (2040-2059 vs 2000-2019) for scenario RCP 4.5 (left), and scenario RCP 8.5 (right).

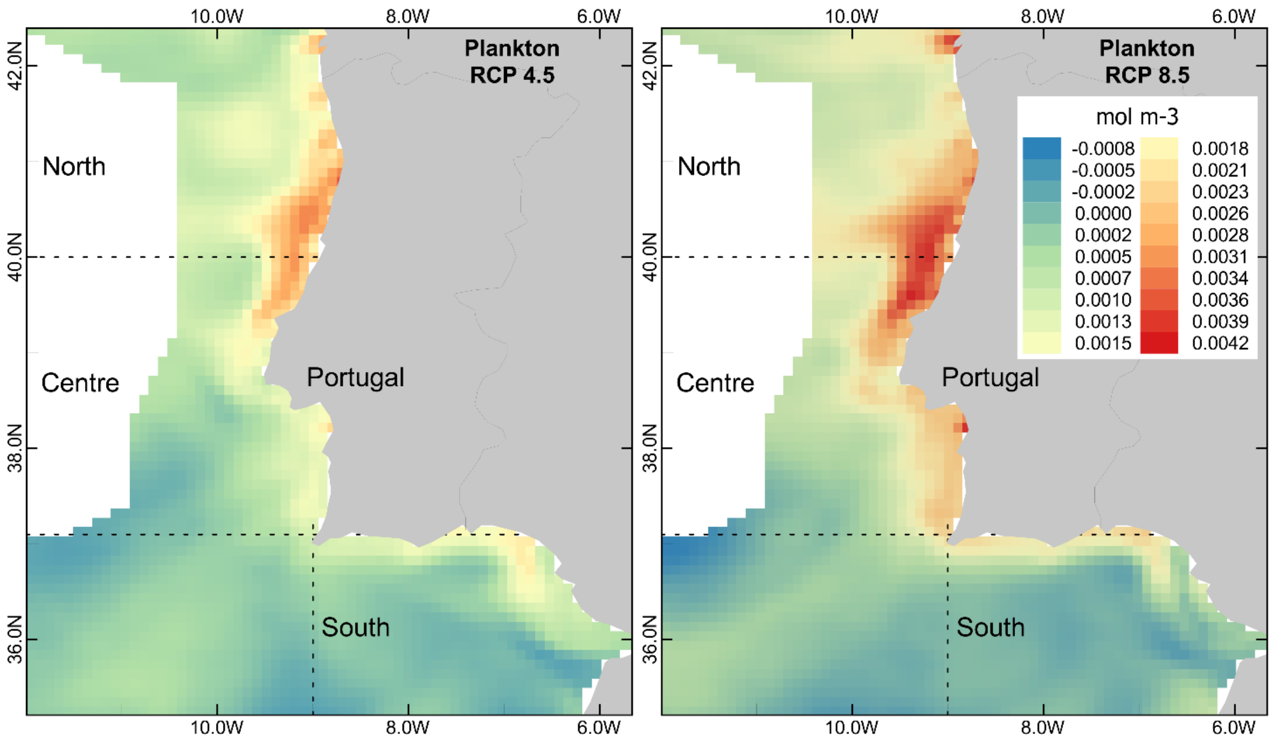

Figure SI3-6. Surface plankton (zooplankton + phytoplankton) concentration (mol m^{-3}) calculated based on period averages (2040-2059 vs 2000-2019) for scenario RCP 4.5 (left), and scenario RCP 8.5 (right).

Bibliography

- Bakun, A.; Black, B. A.; Bograd, S. J.; García-Reyes, M.; Miller, A. J.; Rykaczewski, R. R.; Sydeman, W. J. (2015) Anticipated effects of climate change on coastal upwelling ecosystems. Current Climate Change Reports 1: 85-93.
