# Supplementary material for: Climate change vulnerability assessment of the main marine commercial fish and invertebrates of Portugal
Source: Sci Rep. 2021 Feb 3;11:2958. doi: 10.1038/s41598-021-82595-5 (PMC7858592; doi:10.1038/s41598-021-82595-5)
Supplement: Supplementary file 5 — Supplementary Information 5. [file 41598_2021_82595_MOESM5_ESM.pdf]

Climate change vulnerability assessment of the main marine commercial fish and invertebrates of Portugal

SUPPLEMENTARY INFORMATION 5:

Bootstrap analysis

Juan Bueno-Pardo[1*], Daniela Nobre[1], João N. Monteiro[1], Pedro M. Sousa[1], Eudriano F. S. Costa[1], Vânia Baptista[1], Andreia Ovelheiro[1], Vasco M. N. C. S. Vieira[2], Luís Chícharo[3], Miguel Gaspar[4], Karim Erzini[1], Susan Kay[5], Henrique Queiroga[6], Maria A. Teodósio[1], Francisco Leitão[1]

[1] Centro de Ciências do Mar (CCMAR), Universidade do Algarve, Campus de Gambelas, Faro 8005-139, Portugal

[2] Instituto Superior Técnico, Lisboa 1041-001, Portugal

[3] Faculdade de Ciência e Tecnologia, Universidade do Algarve, Campus de Gambelas, Faro 8005-139, Portugal

[4] Instituto Português do Mar e a Atmósfera (IPMA), Centro de Olhão, Olhão 8700-305, Portugal

[5] Plymouth Marine Laboratory, Prospect Place, The Hoe, Plymouth PL1 3DH, UK

[6] Departamento de Biologia e Centro de Estudos do Ambiente e do Mar (CESAM), Universidade de Aveiro, Campus Universitário de Santiago, Aveiro 3810-193, Portugal

* Corresponding author: jbuenopardo@gmail.com

A bootstrap analysis was carried out to assess the confidence level on the experts evaluations. These analyses consisted on sampling with replacement 15 tallies for each indicator of exposure, sensitivity, adaptive capacity and data quality and calculating the new vulnerability, probability of distribution change and data quality over 10,000 iterations. Here we show the results from these analyses. From the 10,000 iterations, we counted how many times a given species vulnerability, probability of distribution change or directional effects felt into the same category bin: <0.2 (very low), 0.2-0.4 (low), 0.4-0.6 (moderate), 0.6-0.8 (high), >0.8 (very high) (see methods in the main text for more detail).

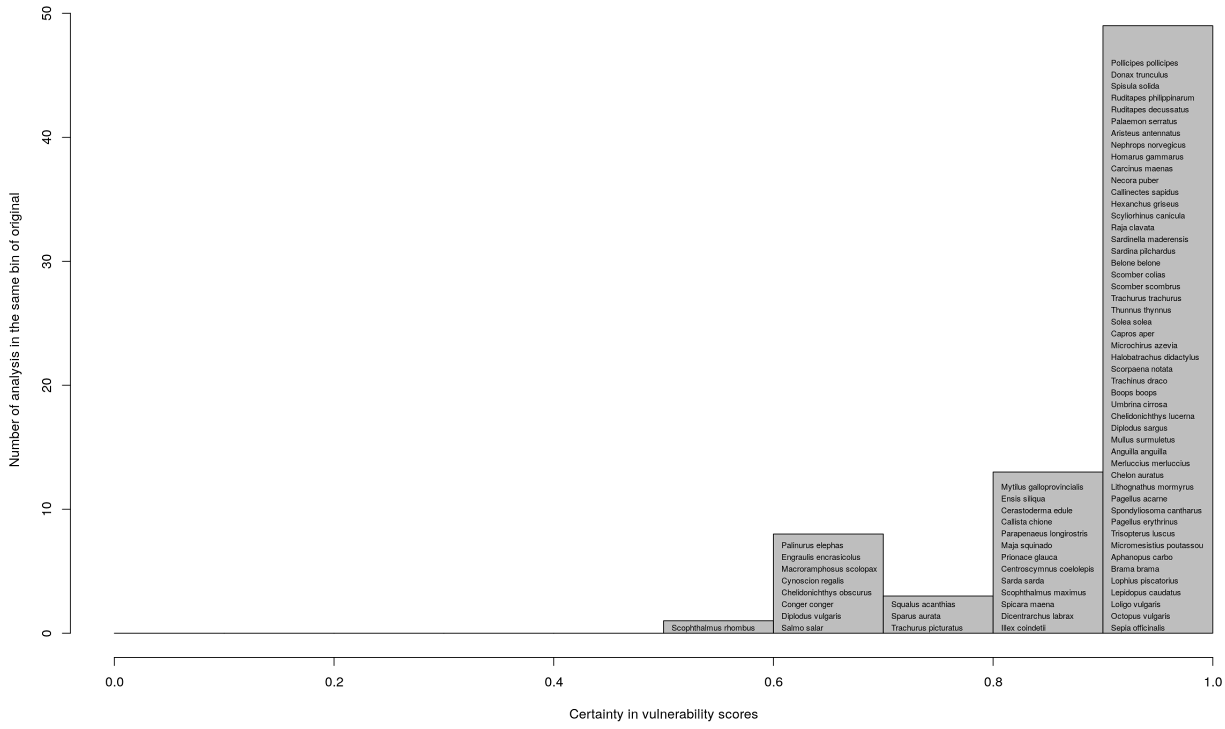

Figure S5-1. Results from the bootstrap analysis for the overall vulnerability assessment.

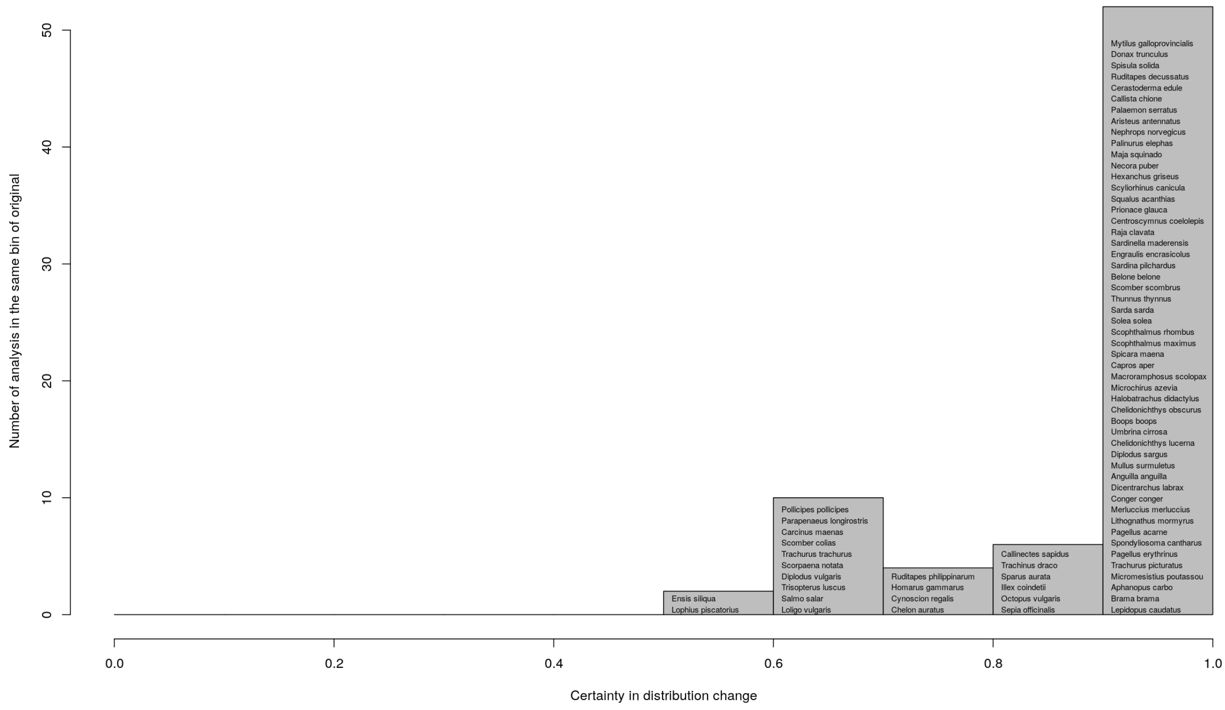

Figure S5-2. Results from the bootstrap analysis for the probability of distribution change assessment.

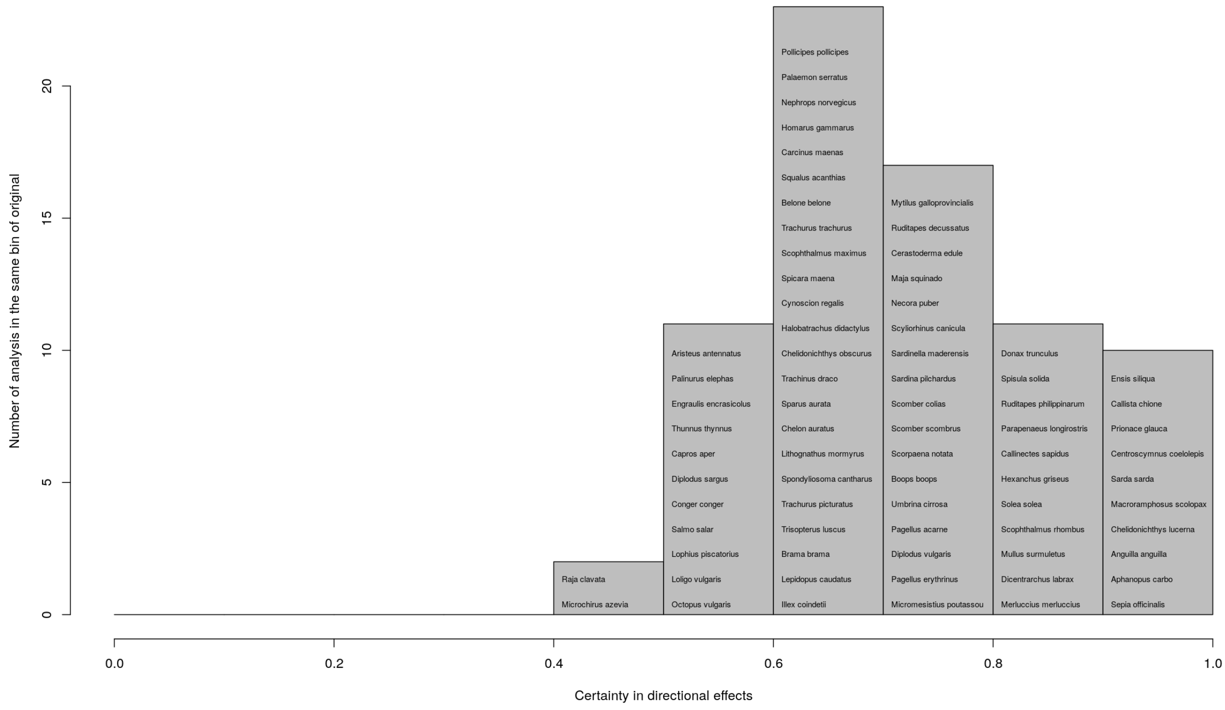

Figure S5-3. Results from the bootstrap analysis for the directional effects assessment.
