# Supplementary material for: Climate change vulnerability assessment of the main marine commercial fish and invertebrates of Portugal
Source: Sci Rep. 2021 Feb 3;11:2958. doi: 10.1038/s41598-021-82595-5 (PMC7858592; doi:10.1038/s41598-021-82595-5)
Supplement: Supplementary file 6 — Supplementary Information 6. [file 41598_2021_82595_MOESM6_ESM.pdf]

Climate change vulnerability assessment of the main marine commercial fish and invertebrates of Portugal

SUPPLEMENTARY INFORMATION 6:

Climate change vulnerability assessment under the RCP 4.5 scenario

Juan Bueno-Pardo[1*], Daniela Nobre[1], João N. Monteiro[1], Pedro M. Sousa[1], Eudriano F. S. Costa[1], Vânia Baptista[1], Andreia Ovelheiro[1], Vasco M. N. C. S. Vieira[2], Luís Chícharo[3], Miguel Gaspar[4], Karim Erzini[1], Susan Kay[5], Henrique Queiroga[6], Maria A. Teodósio[1], Francisco Leitão[1]

[1] Centro de Ciências do Mar (CCMAR), Universidade do Algarve, Campus de Gambelas, Faro 8005-139, Portugal

[2] Instituto Superior Técnico, Lisboa 1041-001, Portugal

[3] Faculdade de Ciência e Tecnologia, Universidade do Algarve, Campus de Gambelas, Faro 8005-139, Portugal

[4] Instituto Português do Mar e a Atmósfera (IPMA), Centro de Olhão, Olhão 8700-305, Portugal

[5] Plymouth Marine Laboratory, Prospect Place, The Hoe, Plymouth PL1 3DH, UK

[6] Departamento de Biologia e Centro de Estudos do Ambiente e do Mar (CESAM), Universidade de Aveiro, Campus Universitário de Santiago, Aveiro 3810-193, Portugal

* Corresponding author: jbuenopardo@gmail.com

The results of the vulnerability assessment under climate change scenario RCP 4.5 are shown. Results from the simulation using RCP 8.5 environmental projections are shown in the main text.

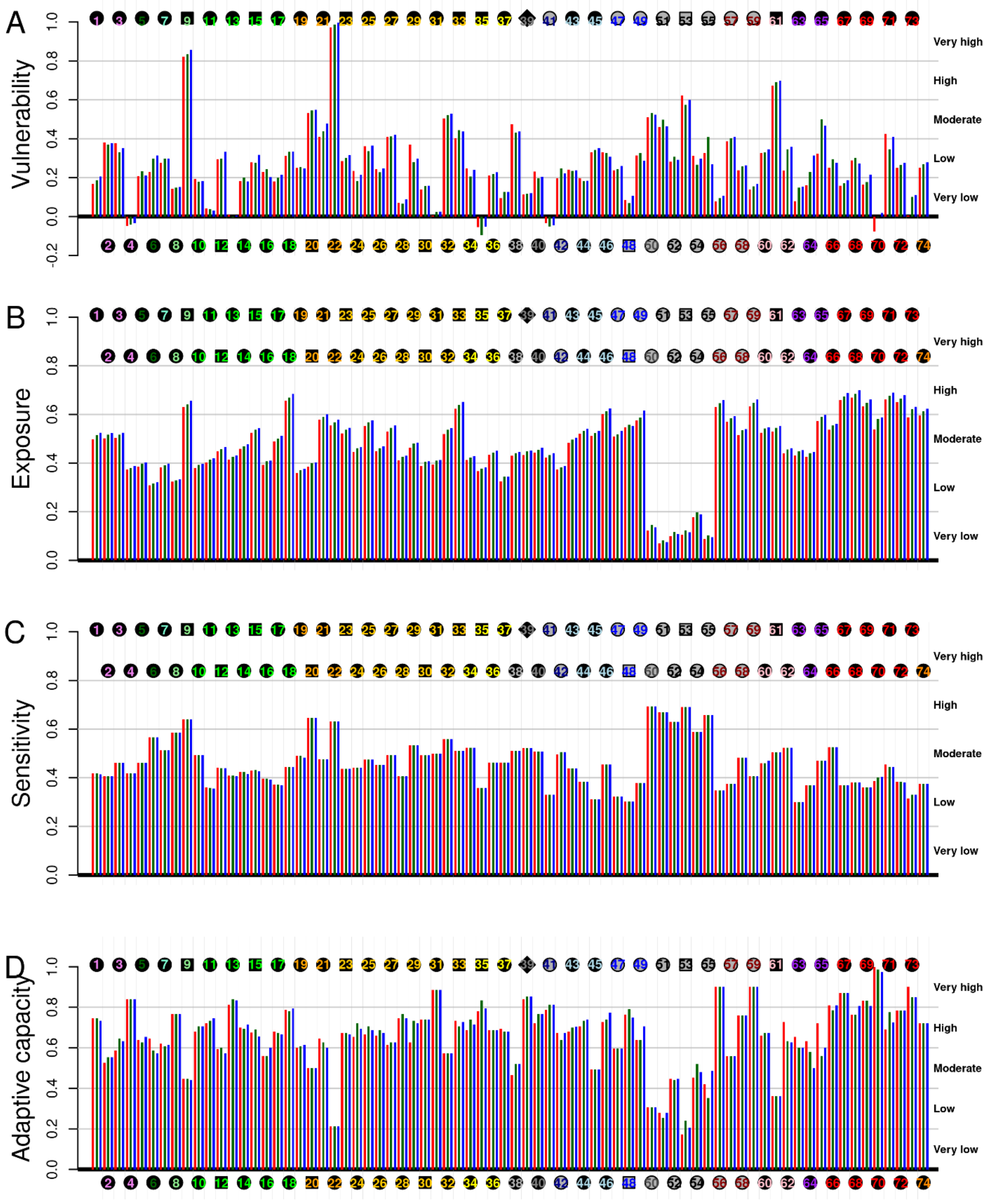

Figure S6-1. Results from the vulnerability analysis in under climate change scenario RCP 5.5. Species number are shown in Table 1 of the main text.
