# Supplementary material for: Climate change vulnerability assessment of the main marine commercial fish and invertebrates of Portugal
Source: Sci Rep. 2021 Feb 3;11:2958. doi: 10.1038/s41598-021-82595-5 (PMC7858592; doi:10.1038/s41598-021-82595-5)
Supplement: Supplementary file 7 — Supplementary Information 7. [file 41598_2021_82595_MOESM7_ESM.pdf]

Climate change vulnerability assessment of the main marine commercial fish and invertebrates of Portugal

SUPPLEMENTARY INFORMATION 7:

Relationship between overall vulnerability, directional effects and indicators

Juan Bueno-Pardo[1*], Daniela Nobre[1], João N. Monteiro[1], Pedro M. Sousa[1], Eudriano F. S. Costa[1], Vânia Baptista[1], Andreia Ovelheiro[1], Vasco M. N. C. S. Vieira[2], Luís Chícharo[3], Miguel Gaspar[4], Karim Erzini[1], Susan Kay[5], Henrique Queiroga[6], Maria A. Teodósio[1], Francisco Leitão[1]

[1] Centro de Ciências do Mar (CCMAR), Universidade do Algarve, Campus de Gambelas, Faro 8005-139, Portugal

[2] Instituto Superior Técnico, Lisboa 1041-001, Portugal

[3] Faculdade de Ciência e Tecnologia, Universidade do Algarve, Campus de Gambelas, Faro 8005-139, Portugal

[4] Instituto Português do Mar e a Atmósfera (IPMA), Centro de Olhão, Olhão 8700-305, Portugal

[5] Plymouth Marine Laboratory, Prospect Place, The Hoe, Plymouth PL1 3DH, UK

[6] Departamento de Biologia e Centro de Estudos do Ambiente e do Mar (CESAM), Universidade de Aveiro, Campus Universitário de Santiago, Aveiro 3810-193, Portugal

* Corresponding author: jbuenopardo@gmail.com

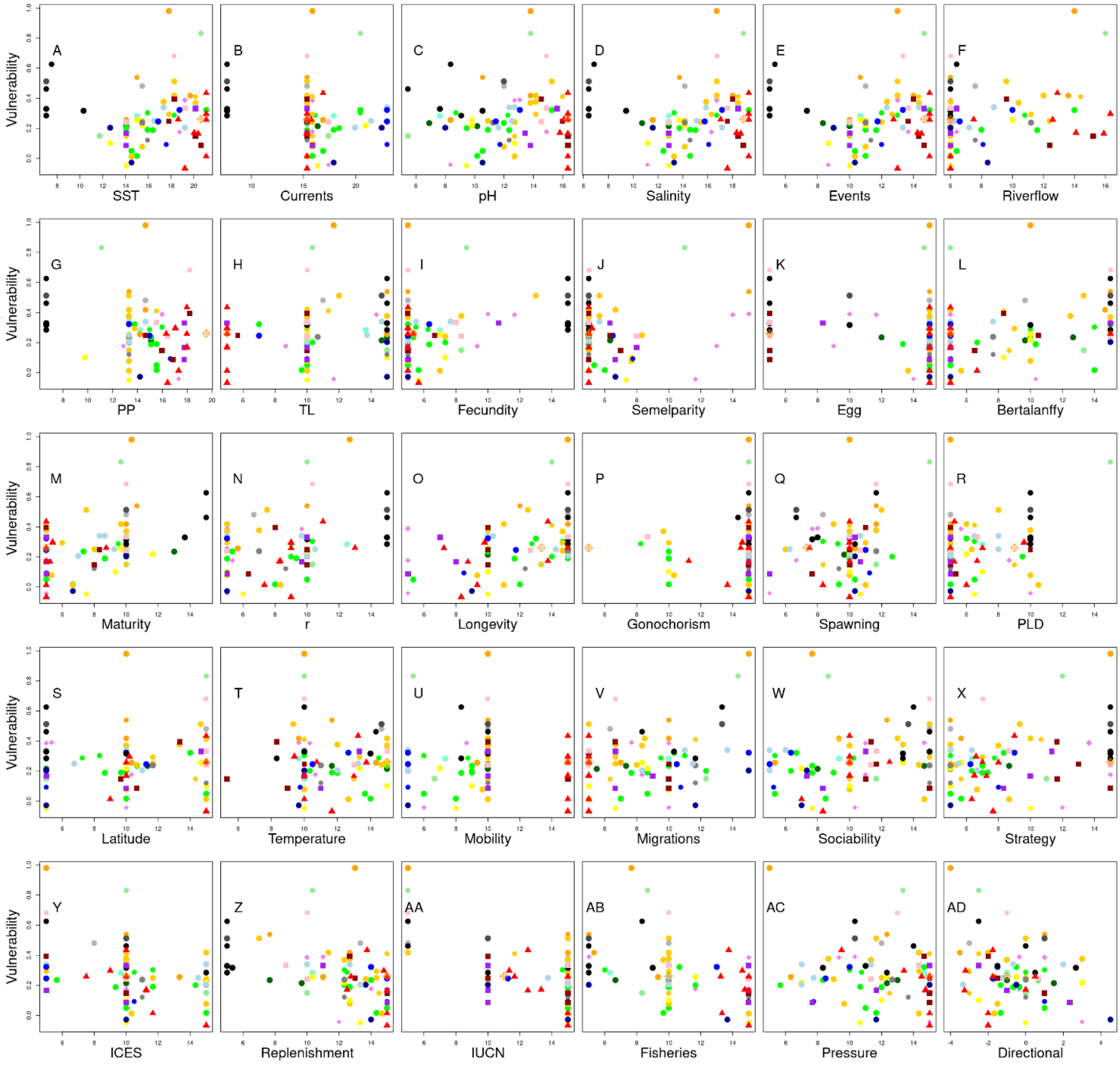

Figure S7-1. Relationship between the overall score of vulnerability and the different indicators used for the assessment of exposure (panels A-G) sensitivity (panels H-X), adaptive capacity (panels Y-AC), and directional effects (panel AD) of the 73 species under consideration. See Fig. 4A for colour and symbol legends and Supplementary Information 3 for the description of the indicators. For each indicator, the average score obtained by the votings of the three experts (5 tallies * 1, 2, or 3 points * weight exposure factor) is provided in the North under scenario RCP 8.5.

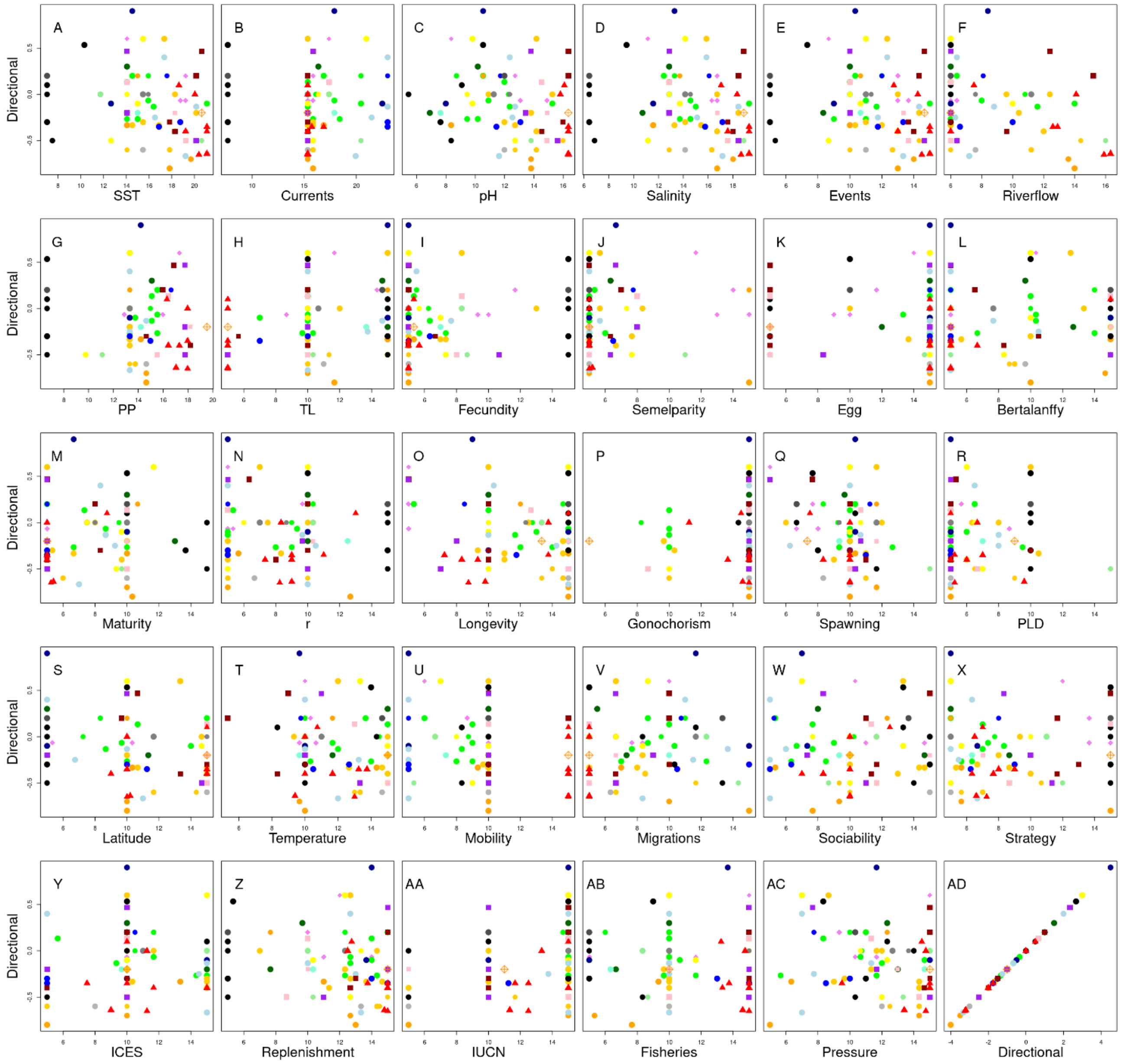

Figure S7-2. Relationship between the directional effects of climate change and the different indicators used for the assessment of exposure (panels A-G) sensitivity (panels H-X), adaptive capacity (panels Y-AC), and directional effects (panel AD) of the 73 species under consideration. See Fig. 4A for colour and symbol legends and Supplementary Information 3 for the description of the indicators. For each indicator, the average score obtained by the votings of the three experts (5 tallies * 1, 2, or 3 points * weight exposure factor) is provided in the North under scenario RCP 8.5.
